# Monpa, memory, and change: an ethnobotanical study of plant use in Mêdog County, South-east Tibet, China

**DOI:** 10.1186/s13002-020-0355-7

**Published:** 2020-01-30

**Authors:** Shan Li, Yu Zhang, Yongjie Guo, Lixin Yang, Yuhua Wang

**Affiliations:** 1 Department of Economic Plants and Biotechnology, Key Laboratory of Economic Plants and Biotechnology, Kunming Institute of Botany, Chinese Academy of Sciences, Kunming, 650201 China; 2 Key Laboratory for Microbial Resources of the Ministry of Education, Yunnan Institute of Microbiology, School of Life Sciences, Yunnan University, Kunming, 650091 China; 3 Germplasm Bank of Wild Species of China, Kunming Institute of Botany, Chinese Academy of Sciences, Kunming, 650201 Yunnan China; 4 University of Chinese Academy of Sciences, Beijing, CN-100049 China

**Keywords:** Indo-Burma biodiversity hotspot, endemic plants, Monpa, cultural change

## Abstract

**Background:**

Due to their relative isolation, the previous studies of Monpa plant use were only conducted in north-east India. In October 2013, Mêdog County was no longer remote, thanks to completion of a highway into the county. This study of plant species used by the Monpa had three research objectives. These were (i) to identify and record local names and uses of plants in Mêdog County, (ii) to assess which of these were uses of endemic or near-endemic species within this part of the Indo-Burma biodiversity hotspot, and (iii) to assess how plant uses reflect socio-economic change in Mêdog County?

**Methods:**

Field surveys were conducted in 12 villages of four townships in Mêdog County, Tibet, China. Two field visits were made. The first field trip was in November 2017 and the second field trip was in May 2018. We interviewed 64 key informants between 21 and 84 years old. Most of them were the village leaders and other local people who are knowledgeable about plants. After transect walks with knowledgeable local people, we used free listing, key informant interviews, and semi-structured interviews during the field work. Plants traditionally used by the Monpa were documented. Utilization frequency was used to assess the significance of each species, and the Cultural Importance index was used to estimate the cultural significance of the species in common. We also used the informant consensus factor (FIC) to determine the homogeneity of the informants’ knowledge of medicinal plants.

**Results:**

One hundred ninety-four plant species belonging to 82 families and 158 genera were recorded and collected. One hundred twenty-two species, primarily fruits, were food plants. Forty-five species were used as traditional medicines. This included highly valued species collected in alpine areas (*Paris polyphylla*) and brought to villages in Mêdog, which are at a lower altitude (between 728 and 1759 m a.s.l). Seven edible plant species were also used as herbal medicines. We also recorded 39 species used for other purposes in Monpa daily life. These included nine species that were used to make agricultural tools, five species for dyes and mordants, four species for timber, three species for fuelwood, four species for religious ritual use, three species for washing, two species for incense, two species for thatching, two species for fiber (rope and paper), two “calendar plants” were used to indicate seasons for agricultural purposes, two fish poison plant species, and one species were used as a tobacco substitute. Based on taxonomic insights and from studies elsewhere, we suggested that fiber species were under-reported (c. 14 species were used vs. one species reported used). Even though these plant species are rich and diverse, the use of endemic or near-endemic species was rarely recorded in previous studies. These species included *Arenga micrantha* (used for starch), *Hornstedtia tibetica* (fruits), *Castanopsis clarkei* (edible nuts) and *Gnetum pendulum* (edible nuts), *Ophiorrhiza medogensis* (vegetables), *Derris scabricaulis* (fish poison), *Radermachera yunnanensis* (agricultural tools), *Litsea tibetana* (seed oil), *Dendrocalamus tibeticus* (wine strainers and implements for administering medicine), *Zanthoxylum motuoense* (spices), *Cinnamomum contractum* (tobacco substitutes), *Morus wittiorum* (medicines), and *Garcinia nujiangensis* (funeral rituals). Despite the absence of roads until 2013 and the impression of “isolation,” Monpa knowledge of plant use reflects three categories of change. Firstly, oral histories of plants used in Bhutan were also encountered by Monpa people after their migration from Bhutan to south-eastern Tibet. Secondly, a “slow change” through centuries of exchange of knowledge (for example of Chinese and Tibetan medical systems), seeds of introduced crops (finger millet (indigenous to Africa), maize (from Meso-America)), and experimentation and use of introduced medicinal plants (such as *Datura stramonium*, which originates from North America). Thirdly, “fast change” over the past decade. This is reflected in changes in traditional architecture and in rising commercial trade in selected plant resources such as *Dendrobium* orchid stems and *Paris polyphylla* rhizomes which are in demand in China’s Traditional Chinese Medicine (TCM) markets).

**Conclusions:**

Monpa people in the south-eastern Tibet have detailed knowledge of the diverse plant resources. But that traditional knowledge is now faced with a crisis because of the modern socio-economic change. In addition, Monpa knowledge of plants reflects slower changes in knowledge as well. For example, Monpa ethnomedicine has been influenced by traditional Tibetan and Chinese medicine over a longer period in time. Overall, this study provides a deeper understanding of the Monpa peoples’ knowledge on wild plants, including endemic and near-endemic species whose uses have not been previously recorded. Several of these narrowly distributed species, such as the fish poison *Derris scabricaulis*, could be the focus of further studies. Some wild edible plants may also have interesting dietary constituents which need in-depth studies. These detailed studies could enable the Monpa people to benefit from the use of their traditional plant-derived culture and therefore support the biodiversity conservation.

## Background

### Introduction

This study was carried out in Mêdog County in the south-eastern Tibet, at the edge of two “biodiversity hotspots” which were the Himalayan and the Indo-Burma biodiversity hotspots. Due to its biogeography, topography, and altitudinal range (a difference of 7500 m over 40 km), south-east area of Tibet has high levels of biodiversity. South-eastern Tibet is also culturally diverse, with Tibetan, Hui, Monpa, Lhoba people developing cultural landscapes through farming, pastoralism, wild plant use, and management. The need to combine traditional ecological knowledge with management strategies to achieve biodiversity conservation in local beliefs and practices is well recognised [1]. In their review of conservation needs for the Indo-Burma biodiversity hotspot, for example, the CEPF (2012) stressed the need for “greatly improved information on status and distribution in Indo-Burma to highlight species for which available information is so limited that it precludes any form of meaningful conservation action”. This study is a contribution toward both conservation and development.

Although a few studies have been done on Monpa plant use in north-east India [2], this is the first study of Monpa ethnobotany in this formerly remote part of China. No longer remote, this area is undergoing rapid socio-economic change, which may lead to declining knowledge of local plant uses. In other parts of the region, however, 68 medicinal plant species were recorded as used by Tibetans in Shangri-la, Yunnan, China [3]. Traditional knowledge of 168 wild edible plant species were recorded in Tibetans of Shangri-la region, Yunnan, China [4]. The uses of 81 species of vascular plants were recorded in Tibetans of Zhouqu county, Gansu, China [5]. The uses of 54 species of vascular plants and 22 mushrooms were recorded in Tibetan community of Zhagana in Tewo County, Gansu, China [6].

### Study aims and research objectives

The aim of this study was to document the ethnobotanical knowledge of the Monpa people in Mêdog County, south-eastern Tibet. As no previous studies had been done on Monpa plant use in China, we had three research objectives. These were (i) to identify and record local names and uses of plants in Mêdog County, (ii) to assess which of these were uses of endemic or near-endemic species within this part of the Indo-Burma biodiversity hotspot, and (iii) to assess how current plant uses reflect socio-economic change in Mêdog County?

## Methods

### Location of the study sites

Mêdog County is the most remote county in the south-eastern Tibet, which covers a total area of approximately 31,395 km^2^, located in the lower river valley of the Yarlung Tsangpo River. It is called “Pe-ma-ko” by the Tibetan Buddhists, meaning “hidden lotus.” The average annual temperature in Mêdog County ranges from 16 to 18 °C. The lowest temperature is 2 °C in January and the highest temperature is 33.8 °C in July. The annual average rainfall is 2350 mm, the frost-free period is about 330 days, and the average humidity is over 80%. In the horizontal distance of 40 km, Mêdog County has an altitudinal difference of more than 7500 m, with plant species ranging from alpine plant species to tropical plant species and complex vegetation structures [7, 8].

We had selected 12 villages belonging to four townships in the Mêdog County from different altitudes for investigation (Table 1). Monpa people accounted for 99.2% of the total population of the Beibeng Township. Dexing Township is the nearest township to the Mêdog County. It is located on the right bank of the Yarlung Tsangpo River, across the river from Mêdog County. Bangxin Township is situated in the northeast of Mêdog County and “Bangxin” which means “flat land” in Tibetan language. Jiaresa Township is located in the northern part of Mêdog County and is one of the three remote towns in the Mêdog County (Table 1).

**Table 1:** Basical geographical information of the Townships

Township	Beibeng	Dexing	Bangxin	Jiaresa
Elevation	400–3260 m	850 m	1240 m	1120 m
Climate	Low mountain tropical humid climate and Mountain subtropical semi-humid climate	Low mountain tropical humid climate and Mountain subtropical semi-humid climate	Mountain subtropical semi-humid climate	Mountain subtropical semi-humid climate
Population	2371	1668	1370	581

**Fig. 1:**
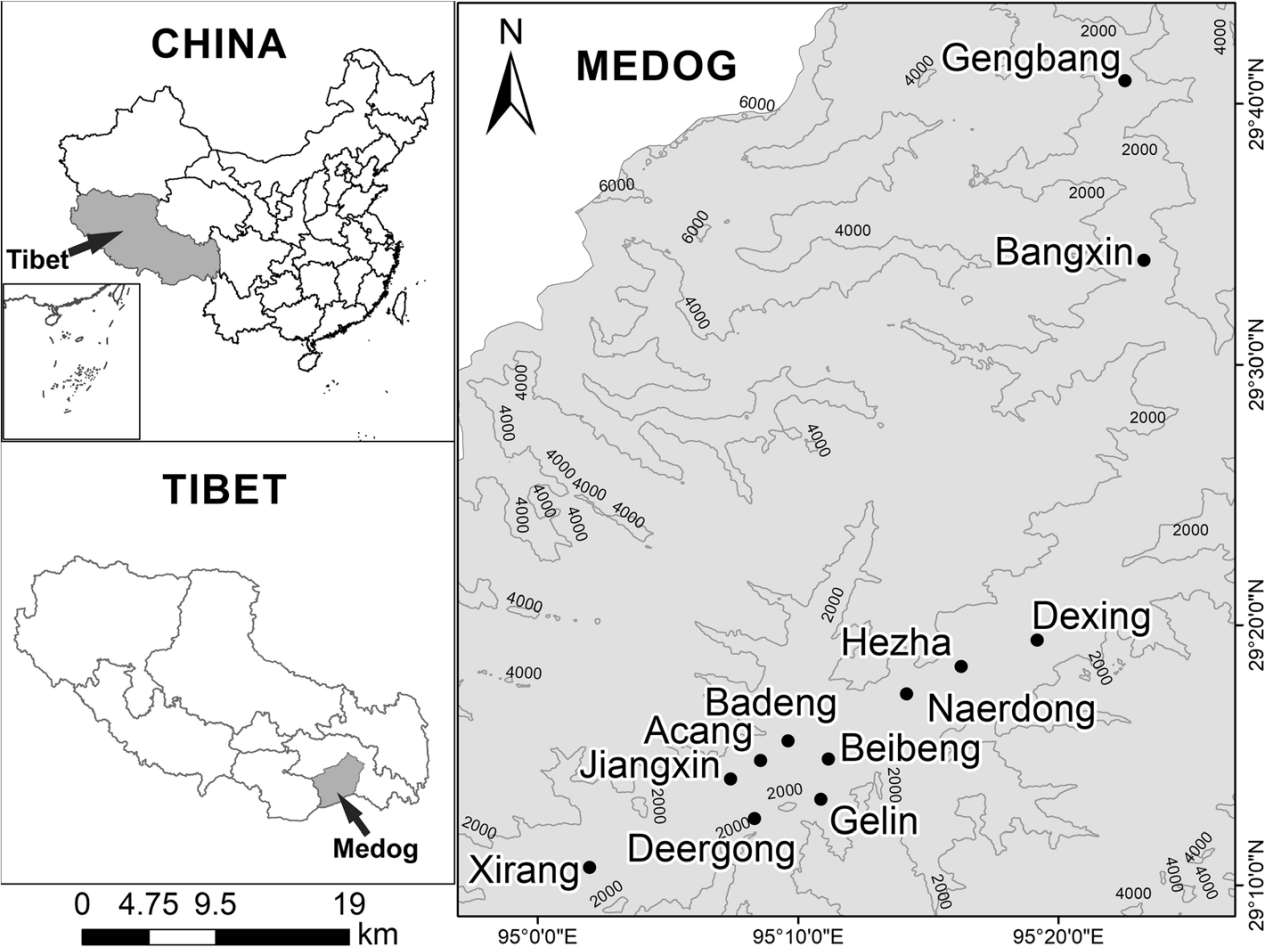
The location of Villages in in Mêdog County, Tibet, China

The location of the 12 study villages in Mêdog County are shown in Fig. 1. The names and altitudes of the seven villages of Beibeng Township are Beibeng Village (839 m a.s.l), Jiangxin Village (893 m a.s.l), Xirang Village (823 m a.s.l), Gelin Village (1759 m a.s.l), De’ergong Village (1552 m a.s.l), Badeng Village (1316 m a.s.l), and Acang Village (1342 m a.s.l). The altitudes of the three villages in Dexing Township are Dexing Village (728 m a.s.l), Hezha Village (1051 m a.s.l), and Naerdong Village (1571 m a.s.l). We also studied one village in Bangxin Township: Bangxin Village (1162 m a.s.l) and one village in Jiaresa Township: Gengbang Village (1330 m a.s.l).

### Cultural background: Monpa in Mêdog

The literal meaning of Monpa is “man of the lower country,” referring to several ethnically related peoples, which may not be related linguistically [9]. In the early to mid-eighteenth century, due to the hardships and heavy taxes in Bhutan at the time, the local Monpa people heard that there was a sacred place in the southeast of Tibet, called Pe-ma-ko (now Mêdog), where there was the chance for a better life, so they decided to flee from Bhutan to Tibet [10]. The oldest record of mentioning Monpa was the Tibetan epic of King Ling Gesar during the fourth century [11]. The language used by “Monpa” belongs to the Tibeto-Burman language group of the Sino-Tibetan language family [9]. However, the Monpa people do not have their own written characters. Consequently, the history of the Monpa people is known through oral histories and Tibetan literature [12]. In 1964, the Monpa group was officially recognized as an independent ethnic group by the People’s Republic of China [13] and approximately 25,000 Monpa currently reside in the low-altitude areas of Tsona, Nyingchi, and Mêdog in the Tibet Autonomous Region of China [14]. In China, the highest population of Monpa speakers lives in Mêdog County, a biologically and culturally diverse region that is 34,000 km^2^ in extent.

### Field survey

Ethnobotanical fieldwork took place over 45 days spreading between November 2017 and May 2018. We interviewed 64 key informants. After transect walks with knowledgeable local people, information was collected through free listing, semi-structured, and key informant interviews, participatory approaches and group discussions. Most of key informants were the village leaders and the knowledgeable persons in the village. The age of key informants ranged between 21 and 84 years old. The interviews included the questions that were relevant to document detail information on all useful wild plants. The questions investigated included: What is your age? What is the local name of each plant? What are the most frequently used parts? What ailment does this plant treated? What is the cooking or preparation method of each plant? Do you know any other uses of the plants? We documented the ethnobotanical information for each plant, including scientific name, vernacular name, parts used, habitat and other specific purposes. Additional file 1 and Additional file 2 local writing system is derived from Tibetan language, so the local name is spelled by the writing system which is founded by Turrell Wylie (https://www.omniglot.com/writing/tibetan.htm) (Additional files 1 and 2). Scientific names of plants were confirmed by The Plant List (http://www.theplantlist.org). All the voucher specimens of listed species were collected and deposited at the herbarium of Kunming Institute of Botany.

### Data analysis

Ethnobotanical quantitative indices including utilization frequency, informant consensus factor (FIC), and cultural importance index (CI) were adopted. The use frequency of certain species was estimated by utilization frequency:

$$ f=\frac{N_m}{N_i} $$

Where *N*_*m*_ was the number of certain species mentioned by informant, and *N*_*i*_ was the total number of informants. High *f* values indicated the plant used frequently [15].

FIC was determined using the following formula to evaluate the information of medicinal plants distributed between informants and to determine the homogeneity of informant’s knowledge on medicinal plants.

$$ \mathrm{FIC}=\frac{N_{ur}-{N}_t}{N_{ur}-1} $$

Where *N*_*ur*_ was the number of use reports from informants for each ailment category, and *N*_*t*_ was the total number of species used by all informants for this ailment category. Values of FIC ranging between 0 and 1. High FIC values (close to one) showed the agreement among the informants about this ailment category. On the contrary, low FIC values (close to zero) showed disagreement among the informants [16].

Each species mentioned by an informant within one use category was a use report (UR). Cultural importance index (CI) was used to indicate the spread of the use (number of informants) of each species as well as to determine diversity of uses.

$$ {\mathrm{CI}}_{\mathrm{s}}=\sum \limits_{u={u}_1}^{u_{NC}}\sum \limits_{i={i}_1}^{i_N}\frac{U{R}_{ui}}{N} $$

*N* was the total number of informants and NC was the total number of use categories. CI was the sum of the proportion of informants that mentioned each of the use categories for a given species. The higher CI value indicated the multiple uses of a species [17].

## Results and discussion

### Diversity and enumeration of Monpa plant use

**Fig. 2:**
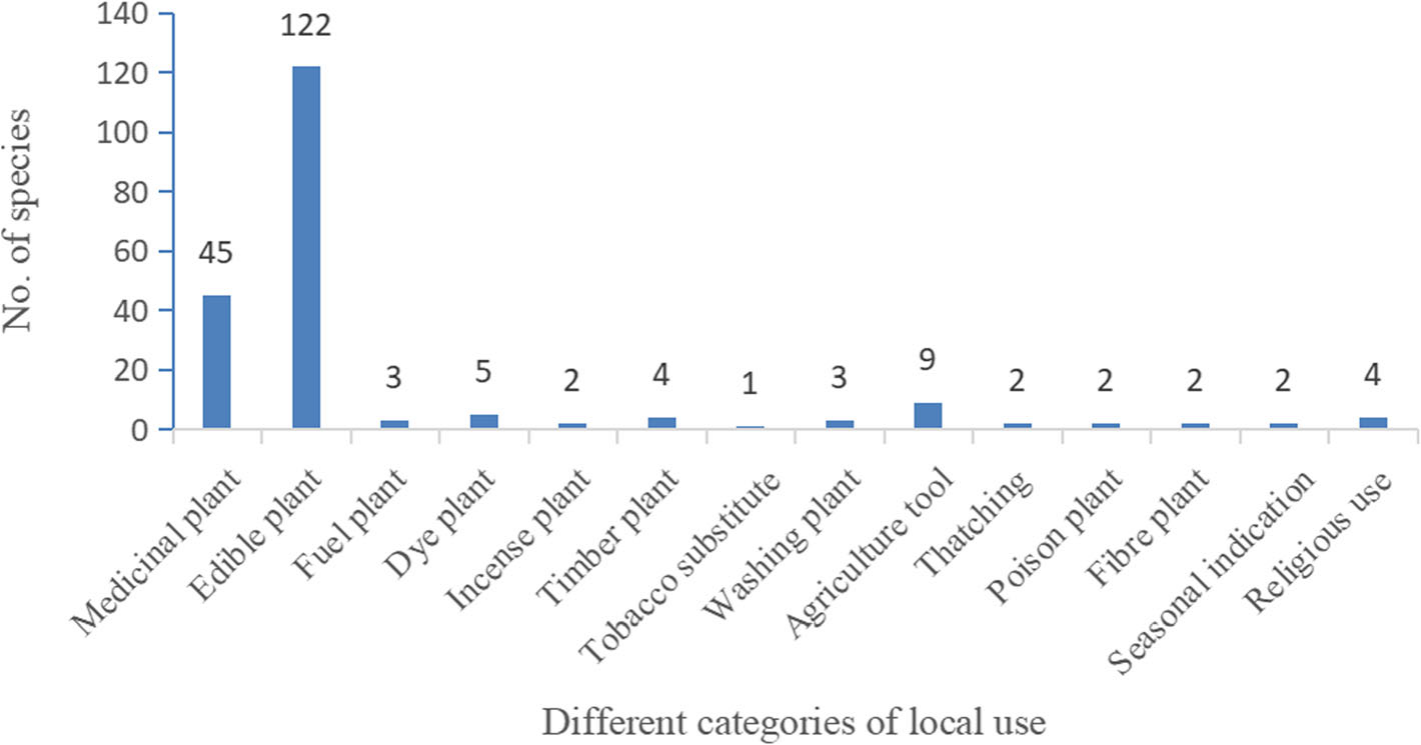
Plants used for different purposes by the Monpa ethnic group in Mêdog County, Tibet, China

This study has documented 194 plant species belonging to 82 families and 158 genera used by Monpa people in Mêdog County (Table 4 in Appendix). Of these plant species, 84 were herbaceous (43.3%), 52 species were trees (26.8%), 35 species were shrubs (18%), and 23 species were lianas or vines (11.9%). The survey results reveal that there are 45 species of ethnomedicinal plants, 122 species of local edible plants, and 39 plant species has been traditionally consumed as other purposes in Monpa daily life, including agriculture tools (9), dyes (5), incense (2), timber (4), fuelwood (3), religious ritual use (4), washing (clothes and hair) (3), thatching (2), fish poisons (2), fiber (2), seasonal indication (2), and a tobacco substitute (1) (Fig. 2).

In contrast to the staple diet of naked barley, yak meat, mutton, buttered tea, and barley wine of traditional Tibetan, they seldom eat vegetables or fruits, yet plant resources in Mêdog County are more plentiful. Monpa people have a wide variety of vegetables and beverages in their traditional daily diet. The most frequently used part is the fruit (58 species, 47.5%) in this study, which is consistent with the situation in northeast India, which has similar bio-climatic conditions [18]. This demonstrates the rich diversity of wild fruits and vegetables in the region, providing local residents with sustainable economic pillars and livelihood security by targeting wild edible fruits and vegetables that can be developed commercially [18]. Out of 122 wild edible species, seven are also used as herbal medicine. *Equisetum ramosissimum*, for example, the root of this species is usually eaten directly as a fruit and boiling the aerial part could be used for treating rheumatism. Wild edible plants with high CI values may have peculiar dietary constituent and require further research. In addition, the alcoholic beverage consumed by almost all Nepalese and Tibetans (known as “jnard”) which has the same ingredients as this yellow “wine” in Mêdog County [19]. Seasonal fishing and fishing by poison are also great economic activities for many tribal people in the world. Studies in Nepal recorded that four entirely different plant species exploited as fish poison plants [20]. Tsering et al.’s study was focused on higher altitude species used by Monpa people, including the medicinal plants *Aconitum heterophyllum*, *Neopicrorhiza scrophulariiflora*, *Paris polyphylla*, *Rhododendron hodgsonii*, *Swertia chirayita*, and *Taxus baccata* [2]. In our study, one of these higher altitude species was recorded (*Paris polyphylla*).

### Edible fruits and vegetables

**Fig. 3:**
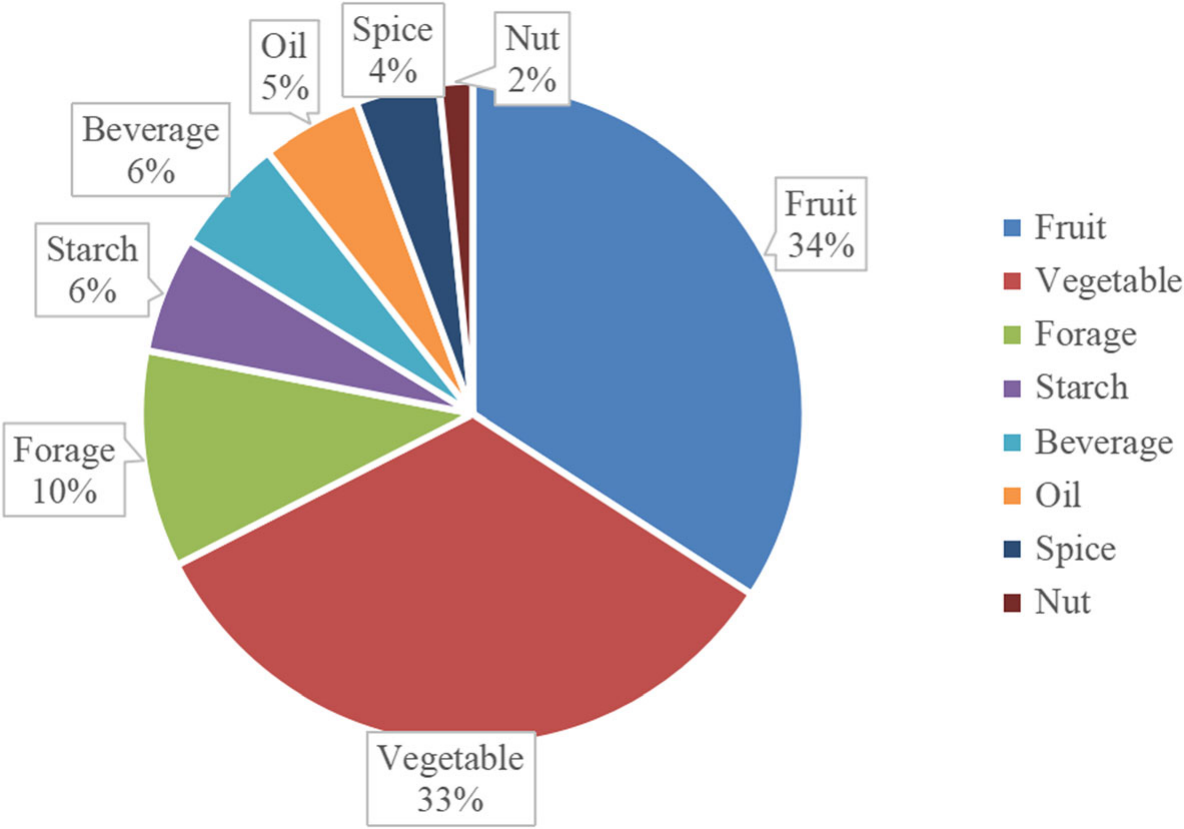
Different categories of edible plants used by the Monpa ethnic group in Mêdog County

Food categories include fruit, vegetable, starch, oil, nut, beverage, condiment, and forage (Fig. 3). The most widely used wild edible species are fruits (42 species), followed by vegetables (41 species). The Monpa depended on wild fruits with high CI values such as *Saurauia punduana* (0.78), *Elaeocarpus braceanus* (0.5), *Duchesnea indica* (0.41), or *Ficus semicordata* (0.39) for vitamins and nutrients. The same as Lhoba people [21], Monpa depended on fruits from wild edible species which may be related to the low productivity of cultivated fruit trees of the Monpa group. *Rubus ellipticus* Sm. (0.33) is a renowned wild edible fruit to Monpa ethnic people in Mêdog County, whose ripe fruits can be taken orally and act as medicine for aperient and juice of the tender leaves cures oral ulcers in the district Udhampur, J&K, India [22]. Monpa people have been using stone casseroles as cookware since ancient times [23]. Monpa people like to eat “hot-pot” dishes in the stone casserole including wild vegetables and meat which are a popular food combination in Mêdog County. Wild vegetables with high CI values included *Crassocephalum crepidioides* (0.78), *Pimpinella diversifolia* (0.56), *Rorippa dubia* (0.31), *Solanum torvum* (0.2), and *Gonostegia hirta* (0.08). *Gonostegia hirta* is a leafy vegetable that is also used as a functional food to provide energy for children and elders [24]. It is interesting that while it is used as a medicine in Bhutan [25], *Entada rheedii* is a popular addition to food by Monpa people. The cooking method for *Entada rheedii* is time-consuming due to the toxicity of the seeds. The seeds have to be detoxified by leaching and heating. The seeds are boiled with water, which is poured off each time to clear away the toxicity, then refilling the pot with water, repeating the process more than ten times. The *Entada rheedii* seeds are then cut into pieces and fried with rice.

Other categories were less frequent in use such as forage (13), starch (seven), beverage (seven), oil (six), spice source plant (five), and nuts (two). However, Monpa people have a rich tradition of extracting beverage, starch, and oil from specific plants in the region.

### Fermented beverages

**Fig. 4:**
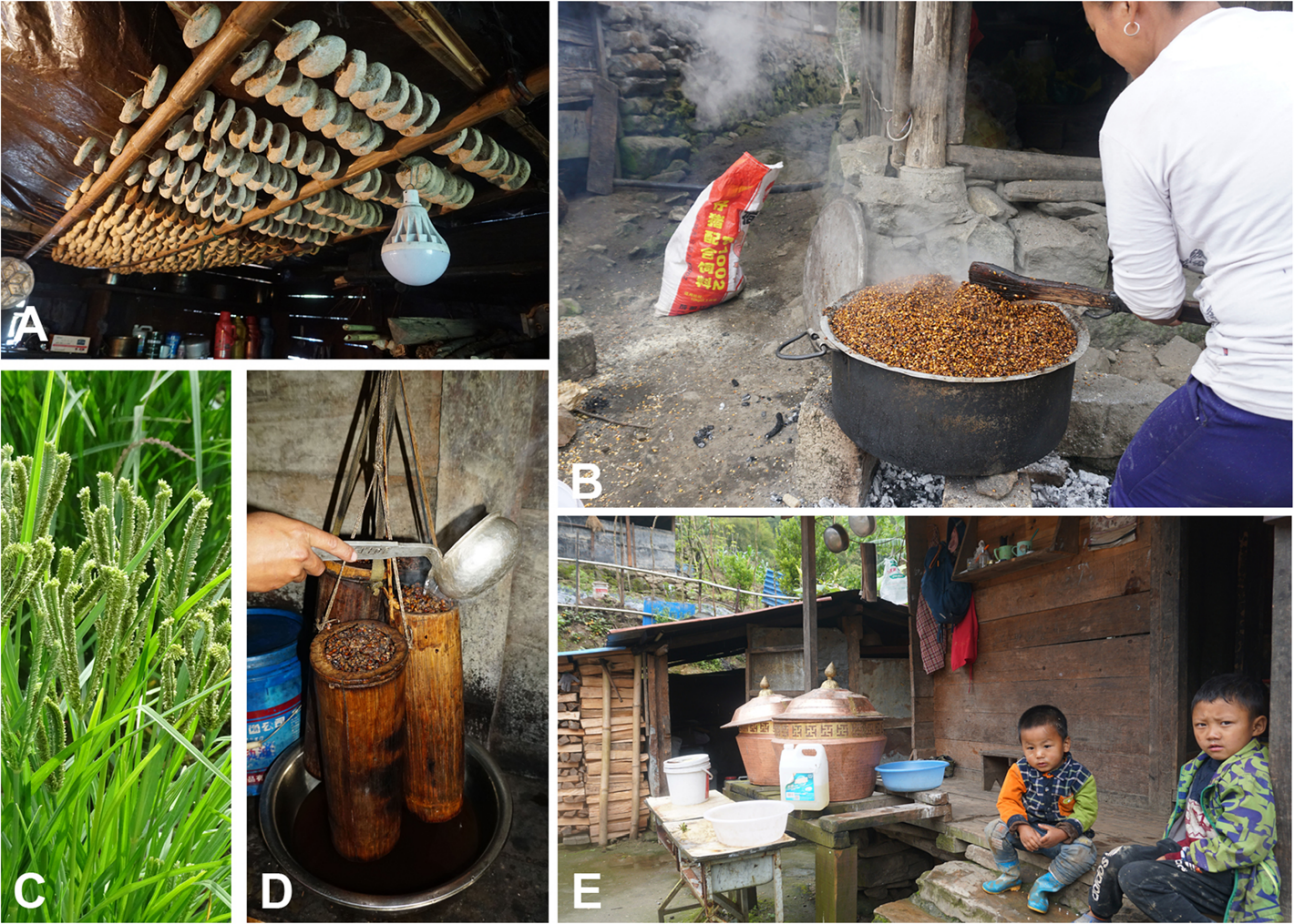
The “yellow-wine” production process. **a** “Cakes” used to start rice “wine” fermentation drying on a bamboo rack. These are made from a mix of species based on a secret recipe. **b** Preparing the starch base for “yellow wine” from rice (*Oryza sativa*), maize (*Zea mays*), *Eleusine coracana*, and *Fagopyrum esculentum*. **c** Finger millet (*Eleusine coracana*). **d** Straining the wine using a strainer made from *Dendrocalamus tibeticus* culms. **e** Ready for a welcome drink of three cups of yellow wine: cultural and social values underpin the continued production of the yellow wine

Traditional consumption of alcoholic beverages is an ancient tradition that is still an integral part of Monpa society. The Monpa people have the traditional custom of “three bowls of wine”, meaning that guests have to drink three bowls of yellow wine before they enter the door to show their friendship. Seven plant species were used to produce a yellow “wine.” The mainly ingredients were rice (*Oryza sativa* L.), maize (*Zea mays* L.), *Eleusine coracana*, and *Fagopyrum esculentum*. *Buddleja asiatica* is the most important plant species during the preparation of alcoholic beverage fermentation by Monpa people in Mêdog County. Based on the uses of *Buddleja lindleyana* and *Buddleja officinalis* in coloring rice yellow [26] and *B*. *officinalis* in indigo fermentation [27], we suggest that *B*. *asiatica* is a dual purposeful species, as both a wine colorant and a source of microorganisms that an aid to fermentation (Fig. 4).

### Starch sources

**Fig. 5:**
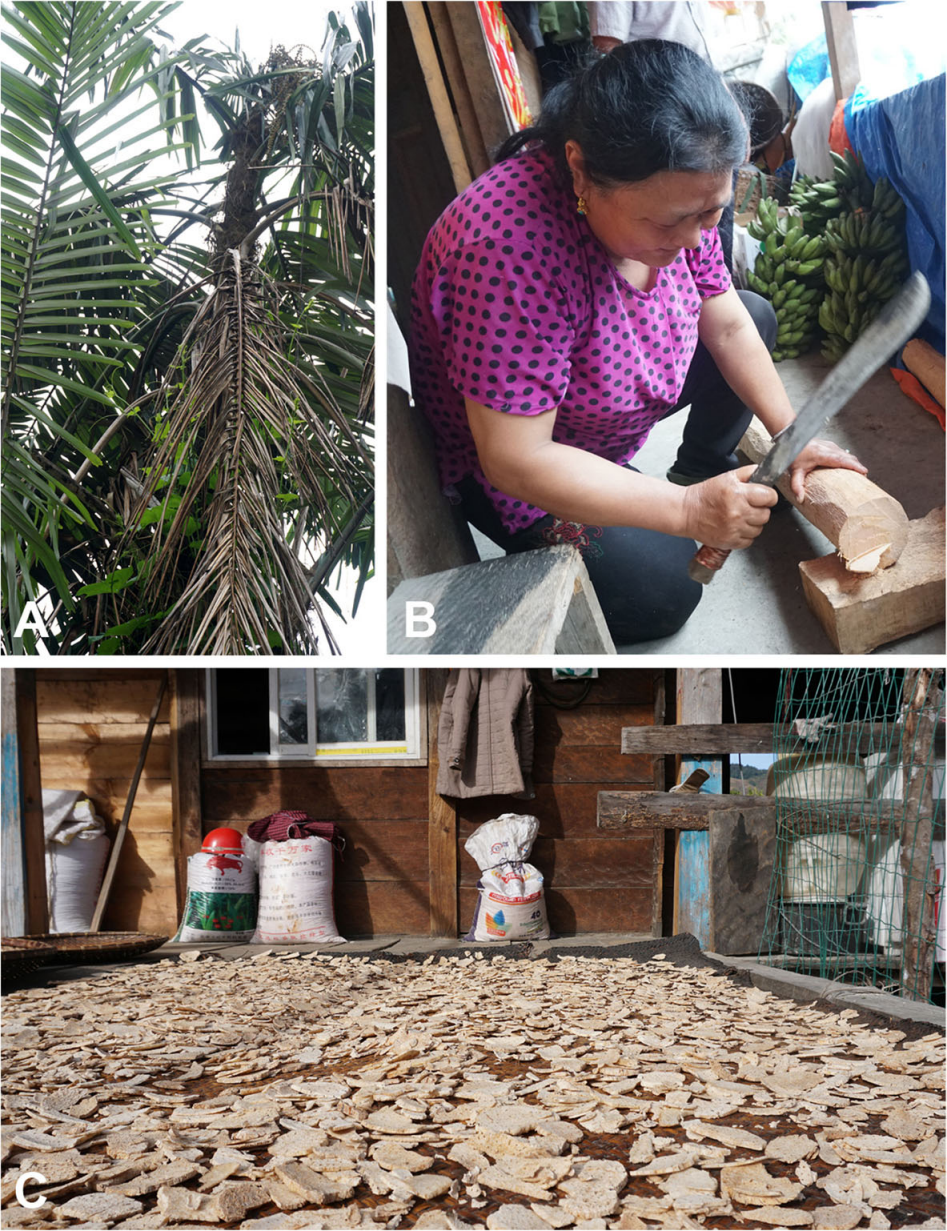
*Arenga micrantha*, a near endemic starch source. **a** *A. micrantha* showing leaves and stems. **b** Slicing the starch rich pith. **c** Slices of *A*. *micrantha* drying before storage

Starch in Monpa people diets was supplemented by starch processed from wild species. Cultivated starch sources were from both cereal crops and cultivated tubers (Table 4 in Appendix). The cultivated cereals were buckwheat (*Fagopyrum esculentum* and *Fagopyrum tataricum*), finger millet (*Eleusine coracana*) rice, and maize. Three taro (*Colocasia*) species and four yam (*Dioscorea*) species were cultivated as starch sources (Table 4 in Appendix). Of these, *Dioscorea alata* tubers have 80% starch in dry matter [28] and *Colocasia esculenta* have 70–80% starch in dry matter [29]. Wild species also provided supplementary starch sources (excluding the starch-rich seeds of *Entada rheedii* mentioned in the previous section). Two of these were palms (Table 4 in Appendix) in the genus *Arenga*. As Ellen points out [30], this is one of main starch-producing palm genera used for food in Asia, the other genera being *Borassus*, *Caryota*, *Corypha*, *Eugeissona*, and *Metroxylon*. Starch production from *Arenga micrantha* is poorly known as it is endemic to Mêdog County [8] and is documented in this study (Fig. 5). In contrast, *Arenga pinnata* is widely distributed in Asia, where it is most commonly served as palm sugar [31], but is also used as a starch source [30]. Other starch sources were *Alsophila articulata* and *Chenopodium album*. Today, the uses of *Arenga micrantha*, *Arenga pinnata*, and *Alsophila articulata* are almost abandoned. This decrease in consumption of these wild starch sources has increased the production of cultivated cereal crops in Mêdog County [32].

### Seed oils

The Monpa in Mêdog County totally used six wild edibles as the source of oil and fats. One of these species, *Perilla frutescens*, was also widely used in various tribal groups of the northeast India [33]. Moreover, *Perilla frutescens* oil is rich in natural compounds that could be developed as nutraceuticals and/or phytomedicine [34].

### Medicinal plant use

According to our survey results, 45 plant species are used as herbal medicines for treating 13 different categories of human ailments. Botanical and ethnobotanical information about these plant species include scientific name, family name, vernacular name, part(s) used, the method of preparation, the ailments treated, and voucher specimen number. Just over half medicinal plants were herbs (51.1%). This agrees with reports from the lower elevation of Bhutan that most of the ethnobotanical plants were herbs [25]. The common use of herbs as sources of medicine found in this study were also indicated by studies conducted elsewhere [35, 36]. Leaves (40%) are the most predominantly used parts of these medicinal plants, followed by roots (22.2%), fruits (15.6%), seeds (11.1%), stems (6.7%), whole plant (6.7%), branches (6.7%), and burgeons (4.4%). The preference for leaf has also been recorded among the traditional Tibetan doctors of Mustang district of the north-central part of Nepal [35].

Uses for all illnesses for wild medicinal plants are locally classified into 13 categories (Table 2). These are skin and subcutaneous tissue diseases, circulatory system, immune system, genitourinary ailments, neurological diseases, inflammation, gastrointestinal ailments, endocrine and metabolism disorders, respiratory system disorders, leech bites, snake bites, abortion, musculoskeletal system disorders, and other diseases. FIC results for the 13 illness categories ranged from 0 to 0.75, with the highest for musculoskeletal system disorders (FIC = 0.75; two species, five use-reports), immune system diseases (FIC = 0.67; two species, four use-reports), and respiratory system disorders (FIC = 0.6; three species, six use-reports) (Table 2). One of the important livelihoods of the Monpa is hunting; the highest FIC for musculoskeletal system disorders is related to the damage caused by the accidents.

**Table 2:** Informant consensus factor for traditional medicinal plant use categories

Illness category	Number of taxa (*N*_t_)	Number of use-reports (*N*_ur_)	Informant consensus factor (FIC)
Circulatory system such as high blood pressure, altitude sickness	3	4	0.33
Endocrine and metabolism disorders such as diabetes	1	1	–
Gastrointestinal ailments such as diarrhea, stomach pain, cholecystitis, intestinal worms	6	10	0.44
Genitourinary ailments such as menstrual problems	2	2	0
Immune systerm such as rheumatism	2	4	0.67
Inflammation, suppuration, infective, toothache, sinusitis, clear heat and detoxification	8	17	0.56
Malaria, mosquito and flea repellent, snake bite, leech bite	5	8	0.43
Morning sickness, abortion	2	3	0.5
Musculoskeletal system such as sprain, arthritis	2	5	0.75
Neurology diseases such as epilepsy, acute alcoholic intoxication	4	6	0.4
Others (heat stroke, refreshing, killing insects, rice blast)	4	5	0.25
Respiratory system disorders such as cold, sore throat and stuffy nose	3	6	0.6
Skin and subcutaneous tissue diseases such as wound, bruises, psoriasis, allergy, scar, leprosy, bleeding, bad skin odor	16	28	0.44

An empirical observation on the use of medicinal plants by the Monpa people of Mêdog County study area requires cross-validation with published literature on phytochemical and pharmacological properties of medicinal plants reported in this study to verify their effectiveness. Our literature review of 21 medicinal plant species shows that local uses are generally consistent with known pharmacological properties. And based on a literature review, 11 medicinal plant species had partial uses similar with reported pharmacological properties. To date, no research studies are available on the phytochemical constituents or pharmacological properties of the *Cinnamomum contractum*, *Brassaiopsis hainla*, *Fraxinus floribunda*, or *Zanthoxylum motuoense*.

Literature studies indicated that seven species, namely *Artemisia vestita*, *Coix lacryma*-*jobi*, *Equisetum ramosissimum*, *Oxalis corniculata*, *Persicaria capitata*, *Uncaria rhynchophylla*, and *Uncaria scandens*, were used in Tibetan medicine to treat the same ailments [37]. Four other species (*Curcuma aromatica*, *Dendrobium catenatum*, *Elaeocarpus braceanus*, *Sambucus williamsii*) were possibly the substitutes for *Curcuma longa* L., *Dendrobium nobile* Lindl, *Terminalia chebula* Retz., and *Sambucus adnata* Wall. in Tibetan medicine. And only one species (*Paris polyphylla*) was used for different purpose by the Monpa than in Tibetan medicine.

Comparison of the information on traditional medicinal plant use of Monpa ethnic group with ethnobotanical studies conducted in the lower elevation of Bhutan, which has similar bio-climatic conditions [25], shows that only one wild medicinal plant, *Datura stramonium*, is used in the same for toothache (Table 3).

**Table 3:** Comparison of indigenous plant use and pharmacological properties of reported medicinal plants

Scientific name	Reported phytochemical/pharmacological properties	Indigenous use [38]	Local use agreed with known properties
*Alocasia longiloba* Miq.	Livestock wounds [37]	Anti-infective and treating burns	Yes
*Altingia excelsa* Noronha	Antipyretic, aphrodisiac and carminative, expectorant, anti-inflammatory, and anticatarrh [39]	Refreshing	Partial
*Artemisia vestita* Wall. ex Besser	Clearing deficient heat, invigorating stomach, promoting diuresis, inducing the expulsion of gas from the stomach or intestines [40]	Stomach pain	Yes
*Begonia acetosella* Craib	Invigorate the circulation of blood [37]	Leeches bite	Yes
*Bidens pilosa* L.	Anti-inflammatory, antiseptic, liver-protective, blood-pressure lowering, and hypoglycemic effects [41]	Cold, sore throat, and stuffy nose	Yes
*Brassaiopsis hainla* (Buch.-Ham.) Seem.	No relevant report found	Arthritis	
*Cinnamomum contractum* H. W. Li	No relevant report found	Stomach pain	
*Citrus medica* L.	Antioxidative, anti-inflammatory, and analgesic [42]	Cold	Yes
*Coix lacryma-jobi* L.	Inhibit obesity and reduce blood lipids [43]	High blood pressure	Partial
*Craibiodendron henryi* W. W. Sm.	Antioxidant activities and vasodilator effects [44]	Rice blast	
*Curcuma aromatica* Salisb.	Antioxident, relieving pain and anti-inflammation, contributing flavor, and preventing cancer [45]	Heat stroke, irregular menstruation, and alcoholism	Partial
*Datura stramonium* L.	Ulcers, wounds, anti-inflammation, rheumatism, bruises, fever and toothache [46]	Toothache	Yes
*Debregeasia longifolia* (Burm.f.) Wedd.	Antitumor, rheumatism [47, 48]	Preventing miscarriage, and bruises	No
*Dendrobium catenatum* Lindl.	Enhancing immunity, resisting tumor, nourishing yin and clearing heat, benefiting stomach and promoting body fluid [49]	Cold	Yes
*Dichroa febrifuga* Lour.	Anti-malarial activity [50]	Mosquito repellent	Partial
*Duchesnea indica* (Jacks.) Focke	Anti-inflammatory, clearing heat, detumescence, and detoxification [51, 52]	Detoxification and bruises	Yes
*Elaeocarpus braceanus* Watt ex C. B. Clarke	Anxiety, depression, nerve pain, epilepsy, and migraine [53]	Diarrhea	No
*Equisetum ramosissimum* Desf.	high blood pressure and diabetes [54]	Rheumatism	No
*Fraxinus floribunda* Wall.	No relevant report found	Sprain and sunburn	
*Hovenia acerba* Lindl.	Alcoholism and vomiting [55]	Alcoholism	Yes
*Impatiens arguta* Hook.f. & Thomson	Amenorrhea, abdominal pain, and hemostasis [37]	Stop bleeding	Yes
*Isodon lophanthoides* (Buch.-Ham. ex D.Don) H.Hara	Enteritis, jaundice, hepatitis, laryngopharyngitis, lepromatous leprosy, and ascariasis [38]	Intestinal worms	Yes
*Leycesteria formosa* Wall.	Traumatic bleeding and fracture [37]	Stop bleeding	Yes
*Millettia pachycarpa* Benth.	Anthelminthic, a medication capable of causing the evacuation of parasitic intestinal worms [56]	Killing insects	Yes
*Momordica dioica* Roxb. ex Willd.	Diuretic, laxative, antihypertensive, anti-inflammatory, and analgesic properties [57]	Cholecystitis	Partial
*Morus wittiorum* Hand.-Mazz.	Antioxidant activity and cytotoxicity [58]	Leprosy	Partial
*Mosla dianthera* (Buch.-Ham. ex Roxb.) Maxim.	Allergic disease is involved in many diseases such as asthma, sinusitis, and rheumatoid arthritis [59]	Allergies	Yes
*Nicotiana tabacum* L.	Antitumor, detoxification and anti-inflammatory [60]	Sinusitis	Partial
*Oxalis corniculata* L.	Giddiness, cough, cold, fever, diarrhea, dysentery, antihelmintic [61]	Morning sickness	No
*Paris polyphylla* ssp.	Anticancer, snake bite, parotitis, mastitis, chronic bronchitis, injuries from fractures, as well as to stop bleeding [62]	Diabetes	No
*Persicaria capitata* (Buch.-Ham. ex D.Don) H.Gross	Anti-inflammatory, antibacterial, analgesic, and diuretic [63]	Burns	Yes
*Piper semiimmersum* C. DC.	Platelet aggregation induced by thrombin (IIa) or PAF in rabbit [64]	Altitude sickness and irregular menstruation	No
*Piper sylvaticum* Roxb.	Anthelmintic, antioxidant and hepatoprotective activities and treatment of bronchitis [65]	Anti-inflammatory	Yes
*Ricinus communis* L.	A Laxative, an anti-infective, or an anti-inflammatory drug [66]	Bruises	Partial
*Sambucus williamsii* Hance	Anti-inflammatory, analgesic, fracture healing [67]	Bruises	Yes
*Sauromatum venosum* (Dryand. ex Aiton) Kunth	Mitogenic and anti-proliferative activity [68]	Suppuration	No
*Solanum aculeatissimum* Jacq.	Constipation, back pain, snakebites, toothache, headache, skin infections, cough [69]	Psoriasis	Partial
*Stephania abyssinica* (Quart.-Dill. & A.Rich.) Walp.	Treat various stomach disorders, laxative, antidote, regulator of menstrual cycle [70]	Rheumatism and snake bite	Partial
*Swertia angustifolia* Buch.-Ham. ex D. Don	Febrifuge and epilepsy [71]	Anti-malaria	Yes
*Swertia nervosa* (Wall. ex G. Don) C. B. Clarke	Clearing away heat and toxic material, invigorating blood circulation and regulating menstruation [72]	Diarrhea	Yes
*Toddalia asiatica* (L.) Lam.	Relieve pain and stasis as well as for haemostatic, treat malaria, fever and to cure rheumatism [73, 74]	Epilepsy	No
*Uncaria rhynchophylla* (Miq.) Miq. ex Havil.	Treatment of hypertension, headache, and stroke [75]	High blood pressure	Yes
*Uncaria scandens* (Sm.) Hutch.	Treatment of nosotoxicosis, headache, dizziness, high fever in children, seizures, convulsions [76]	Epilepsy	Yes
*Viburnum cylindricum* Buch.-Ham. ex D. Don	Cough, diarrhea, rheumatoid arthritis, and tumefaction [77]	Anti-inflammatory, scar and repelling flea	Partial
*Zanthoxylum motuoense* C. C. Huang	No relevant report found	Bad skin odor	

### Natural dyes and mordants

*Strobilanthes cusia* (Yang-shar-pa), *Rubia wallichiana* (Lae-nyi), *Rubia membranacea* (Lae-nyi), *Eurya acuminata* (Zem-shing), and *Curcuma longa* (Dgrong) are the commonly used species in traditional dye processes. Fresh stems and leaves of *Strobilanthes cusia* are a well-known indigo source [78]. Boiling the stems of *Rubia membranacea* are used as a red dye. Mashed *Curcuma longa* tubers are used to dye threads yellow, while *Eurya acuminata* is used to dye threads green. In India, *Eurya acuminata* is used as a mordant together with *Rubia cordifolia* [79]. Whether *E*. *acuminata* is used in Mêdog as a mordant with *Rubia membranacea* or *Rubia wallichiana* is unknown at this stage, but is worth further investigation. This is because the genus *Eurya* (Pentaphylacaceae) is a known aluminium hyperaccumulator group [80] that are effective as mordants for red dye processes together with anthroquinone rich dye species, including *Rubia* [81].

### Paper making

The Monpa community in Linzhi city is famous for hand-made paper for religious scripts that is made from the stem bark of *Edgeworthia gardneri*. Peeling the stem bark and removing the outermost layer of the stem bark, the remaining parts are soaked in the water, then stir the solution into a viscous state, pour the solution into a wooden flat mold, and dry it into a paper. Linzhi paper is better than Tibetan paper produced elsewhere.

### Fibers for rope and string

Although *Edgeworthia gardneri* (Thymelaceae) can also be used for making rope and string, the value of this species for paper making may be a reason why this alternative use was not mentioned by local people during our fieldwork. What was mentioned as a source of rope was the aerial roots of *Poikilospermum lanceolatum* (Urticaceae). Based on taxonomic insights and from studies elsewhere, however, we suggested that fiber species were under-reported (c. 14 species in four families (Fabaceae, Moraceae, Urticaceae, and Thymeleaceae) were used compared to the use of just one species (*Poikilospermum lanceolatum*) reported used for rope making). For example, *Debregeasia longifolia* (Urticaceae) is known for the quality of its fibers from other parts of China and *Millettia pachycarpa* (Leguminosae) bark is also recorded used for rope in the Flora of China (www.efloras.org). It is also likely that the bark of Moraceae (*Ficus auriculata*, *Ficus cyrtophylla*, *Ficus oligodon*, *Ficus semicordata*, *Ficus subincisa*, *Morus alba*, and *Morus wittiorum*) are also used for rope or twine. As is the stem bark of several Urticaceae (*Elatostema cuneiforme*, *Elatostema nasutum*, *Gonostegia hirta*, *Pilea hilliana*)*.*

### House construction, tools, and utensils

*Morus wittiorum*, *Celastrus glaucophyllus*, *Terminalia myriocarpa*, and *Pinus wallichiana* are the main timber species that the Monpa used for building their houses, of which *Terminalia myriocarpa* is the best quality of all timber species but cannot be chopped now because of it is vulnerable species according to the China Red Data Book [82]. *Erythrina arborescens*, *Wendlandia tinctoria*, *Maesa rugosa*, *Radermachera yunnanensis*, *Abroma augusta*, *Macaranga denticulata*, and *Phrynium placentarium* are used to make agricultural tools or daily-life utensils. For example, *Wendlandia tinctoria* can be used to make hilts for knives and *Erythrina arborescens* is used to make carvings for religious rituals.

*Imperata cylindrica* and *Themeda villosa* are used for thatching, of which the quality of *Imperata cylindrica* is better than *Themeda villosa*. *Themeda villosa* are covered on the roof, paved 5 cm thick, and changed once in 3years, but *Imperata cylindrica* can be maintained 7 years.

**Fig. 6:**
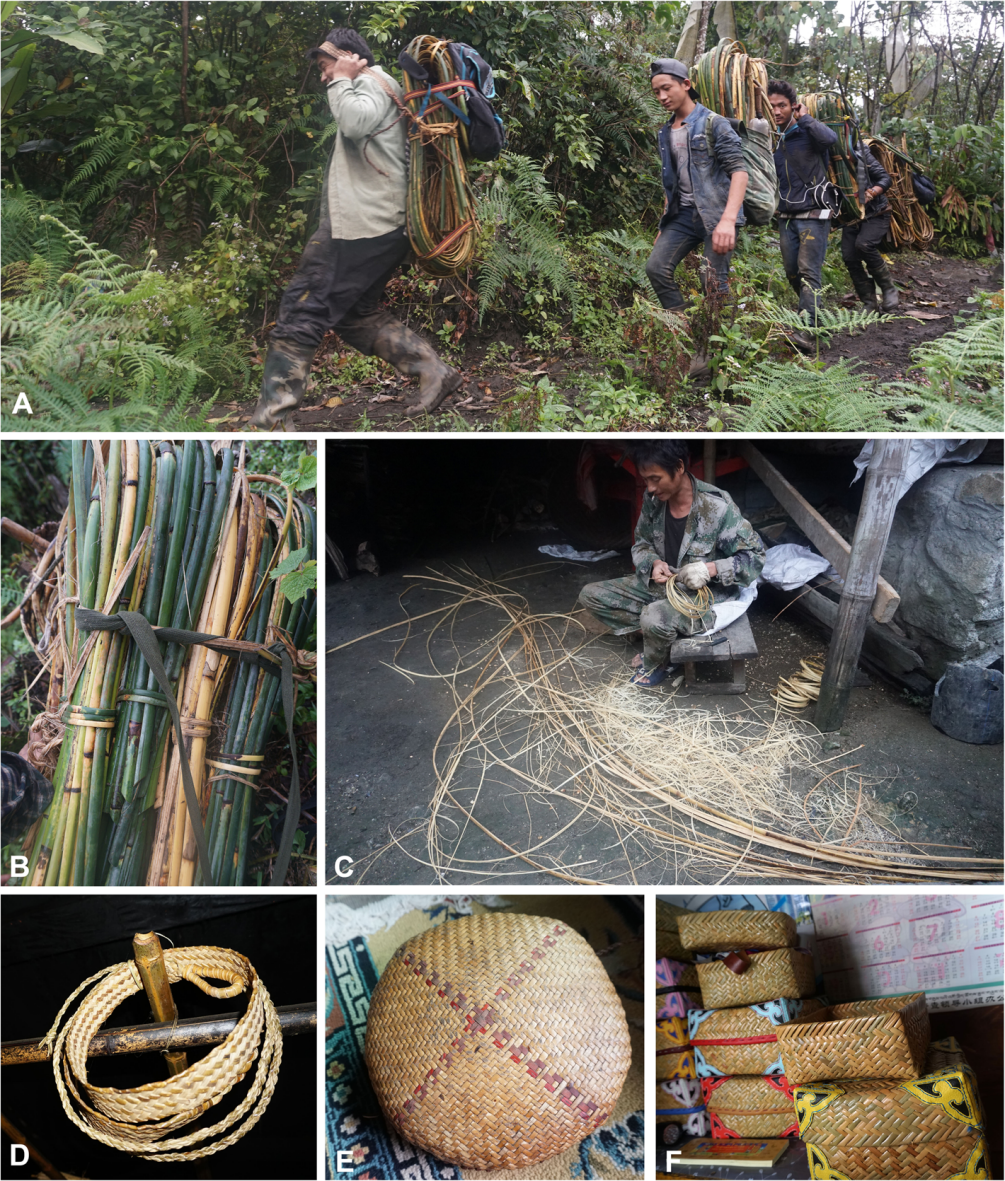
Rattan as a basketry resource. **a** Men returning to their village with bundles of *Calamus acanthospathus* stems. **b** Details of harvested *C. acanthospathus.* **c** Splitting *C*. *acanthospathus* in preparation for weaving. **d** A woven rattan strap for a carrying basket. **e**. Completed winnowing basket. **f** Storage baskets

In addition to use of bamboo (mainly *Bambusa teres* and *Dendrocalamus tibeticus*) for making household utensils, the rattan *Calamus acanthospathus* is used to make baskets (Fig. 6). This widespread species is found in China (Tibet, Yunnan) as well as in Bhutan, India, Laos, Myanmar, Nepal, Thailand, and Vietnam which is also used as a source of edible greens (from the young shoots).

### Ritual uses of plants

The Monpa belief systems, derived from the pre-Buddhist Bon religion and from Tibetan Buddhism, also link to animistic beliefs, where even the new houses have soul [83]. *Laurocerasus undulata* seeds oil and liquor are blended together during religious rituals by Monpa people, and then poured near the house to drive away misfortune and malevolent spirits. Nowadays, Monpa culture has been deeply influenced by Tibetan culture and most of the Monpa people believe in Tibetan Buddhism. *Cinnamomum iners* and *Elsholtzia blanda* are used as incense sources, which play an important role in the religious rituals of the Monpa. *Luculia gratissima* locally called “nom-meng” is also used as an offering in religious rituals.

The Monpa in Mêdog County have a unique funeral culture owing to the special geographical environment, cultural background, and religious beliefs. The practice of re-burial occurs when after burial, the bones are dug out for cremation, and the ashes are scattered into the Yarlung Tsangpo River. This cremation is ignited by the leaves of *Altingia excelsa* locally called “Sang-shing,” which was used by the Monpa people to burn the body. In the meantime, yak butter and liquor are periodically added to the fire, along with offerings of rice, maize, and “Kon-pu” (*Eleusine coracana*). Secondly, put the leaves and stems of “Sra-gu” (*Arundo donax*) and the branches of “La-ga-dong-shing” (*Garcinia nujiangensis*) in turn. The leaves of “Sang-shing” are an essential ignition material for every funeral of the Monpa people.

### Fish poisons

Seasonal fishing and hunting are great economic activities of Monpa ethnic community in addition to agriculture. Fishing by poison was well-known throughout the world in historical time [84]. *Derris scabricaulis* and *Hydrocotyle javanica* are poisonous plants used by the Monpa people in Mêdog County for fishing. A proper amount of poisonous plants are soaked in the water, the fish will lose consciousness and float to the water within half an hour. However, if you could not catch the fish in time, the fish will wake up. No relevant reports about these two poisonous plants were found. However, the rotenones, saponins, and cyanide are the main active ingredients of these fish poison species [85].

### Calendar plants

According to our investigation, *Mussaenda pubescens* and *Meliosma pinnata* are used for indicating the time of sowing maize by Monpa people. *Mussaenda pubescens* blossoms and the weather is fine, then you can sow the seeds in the field, but you cannot sow the seeds after the blooming period of *Meliosma pinnata*. The flowering period of *Mussaenda pubescens* is during June to July. The blooming period of *Meliosma pinnata* is during May to June [86]. The flowering time of these two species is exactly the same as maize planting time.

### Uses of endemic and near-endemic plant species

**Fig. 7:**
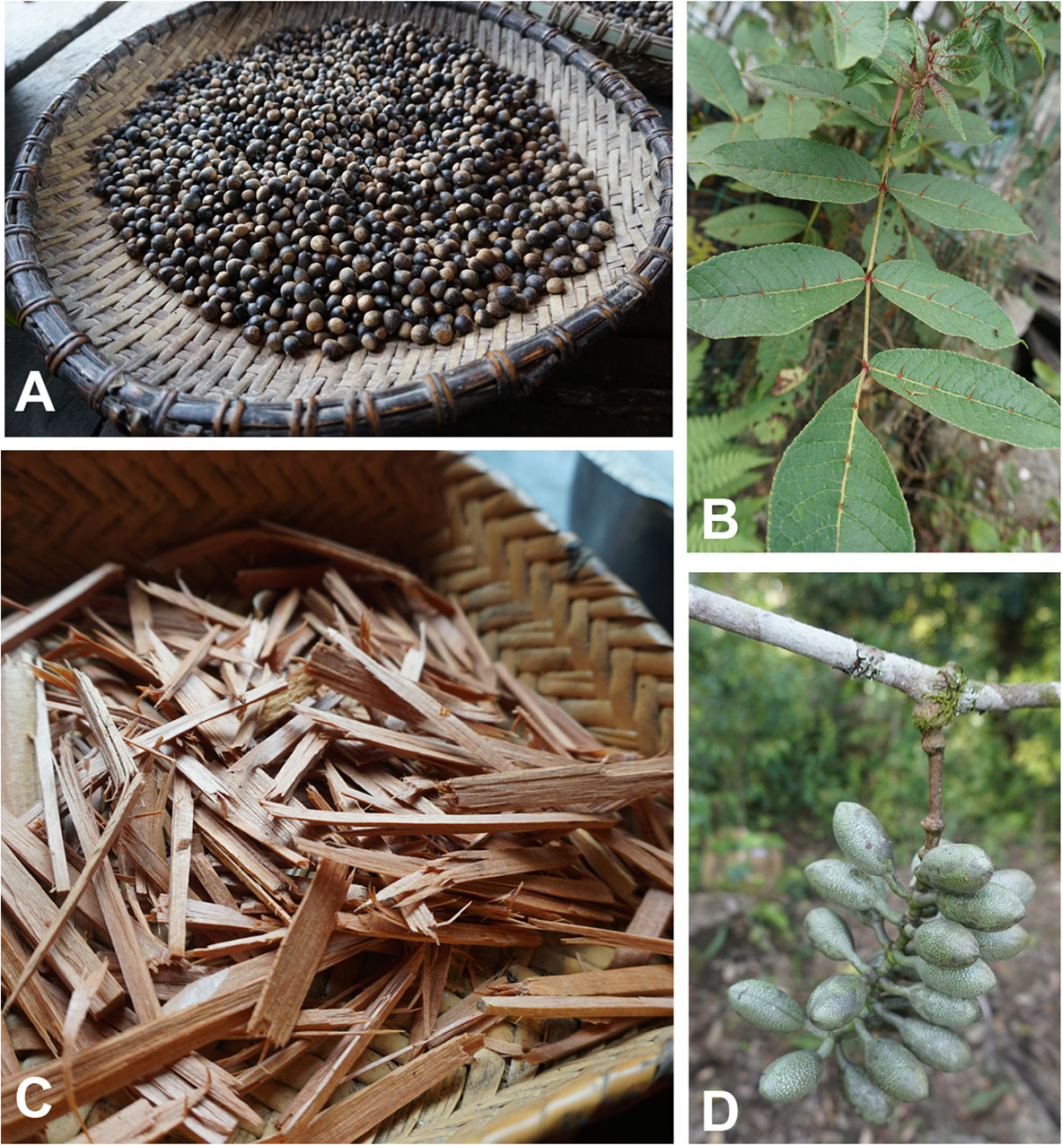
Usual sources of food, spice and incense used by Monpa people. **a** Edible nuts from *Castanopsis clarkei* King ex Hook.f.. **b** *Zanthoxylum motuoense* C. C. Huang, a local source of spicy fruits. **c** *Platycladus orientalis* (L.) Franco wood used for incense. **d** *Gnetum pendulum* C. Y. Cheng., a near-endemic species with edible fruits

Uses of endemic and near-endemic species in 12 plant families reflect the plant diversity of this part of the Indo-Burman biodiversity “hot-spot.” Uses of these species have been rarely recorded in previous studies. In our field survey, we recorded the uses of narrowly distributed edible plants, for example, *Arenga micrantha* (used for starch), *Hornstedtia tibetica* (for fruits), *Castanopsis clarkei*, and *Gnetum pendulum* (for edible nuts) (Fig. 7a, d) and *Ophiorrhiza medogensis* (for vegetables). In contrast to the widespread use of the poisonous *Derris trifoliolata* (Leguminosae) containing rotenone, which occurs from East Africa to the Western Pacific, Monpa people use *Derris scabricaulis* which is endemic to Yunnan and Tibet*.* In terms of farming and equipment used by local households, agricultural tools are made from the high-density wood of *Radermachera yunnanensis* (Bignoniaceae) while wine strainers and implements for administering medicine are made from *Dendrocalamus tibeticus* (Poaceae). *Litsea tibetana* (Lauraceae) is a near endemic seed oil source, as are spices from the endemic *Zanthoxylum motuoense* (Rutaceae). Additional unusual records are the use of *Cinnamomum contractum* (Lauraceae) as a tobacco substitute (a species only found in south-east Tibet and NW Yunnan, *Morus wittiorum* (Moraceae) fruits for medicine and the use of *Garcinia nujiangensis* (Clusiaceae), a species restricted to south-east Tibet, north-west, and west Yunnan for funeral rituals.

### How do plant uses reflect socio-economic change in Mêdog County?

Until October 2013, there were no major roads in Mêdog County and Monpa people practised swidden agriculture, supplemented by hunting and gathering [87]. Despite the absence of roads, Monpa knowledge of plant uses reflect at least three categories of change.

**Fig. 8:**
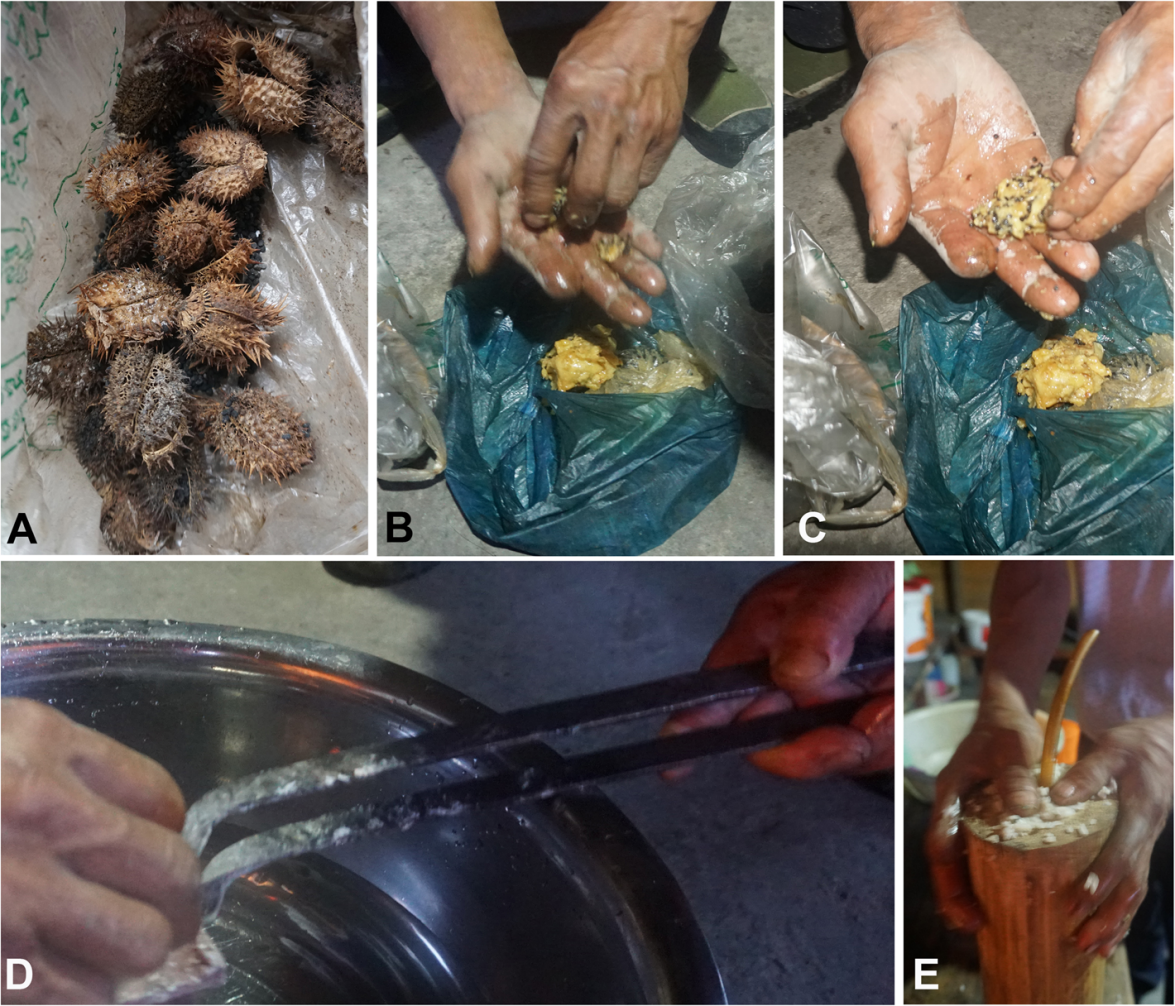
Experimentation and use of an introduced species in Monpa “traditional” medicine. **a** *Datura stramonium* fruits. The seeds are used to treat toothache. **b**, **c** Mixing *D*. *stramonium* seeds with pig fat. **d** Creating hot steam by placing a red hot iron in water, on which the *Datura* seed/fat mixture is placed. **e** *Dendrocalamus tibeticus* bamboo culm, sealed using rice around a protruding tube that is placed over the super-heated *Datura* and pig-fat infused steam to direct the ingredients to the sore tooth and remove the “insect” causing toothache

Firstly, through oral history, knowledge of plants that Monpa ancestors would have encountered in Bhutan and Tibet prior to their migration to Mêdog. Secondly, a slow change in knowledge as introduced species were brought to Mêdog along the trade routes. These species include cereal crops from Africa (*Eleusine coracana* and *Sorghum bicolor*) and meso-America (*Zea mays*), cultivated fruits from north-west China (*Prunus persica*), medicinal plants from Africa (*Ricinus communis*) and North America (*Datura stramonium*), fuel from the Mediterranean Basin and the Middle East (*Arundo donax*), and three South American Solanceae that have come into “traditional” use (*Nicotiana tabacum*, *Solanum americanum* and *Solanum aculeatissimum*). Although oral histories do not indicate when these species were introduced, the fact that several introduced species are used ritually (maize, *Eleusine coracana* and *Arundo donax*) is one indication of early introductions. Another indication is the “traditional” medicinal use of introduced medicinal plants such as *Datura stramonium* (Fig. 8). Although *D*. *stramonium* seeds are known to be used for treating toothache elsewhere [88], but the method of preparation and administration used by Monpa people is innovative (Fig. 8a–d). Thirdly, in contrast to these “slow changes,” there is “fast change” over the past decade that has speeded up rapidly since the highway was opened in October 2013. This is reflected in changes in traditional architecture and in trade in selected plant resources (such as *Dendrobium* and *Paris* to China’s TCM markets).

While Monpa people still have a wealth of ethnobotanical knowledge that has been passed down orally from generation to generation, the construction of a highway to Mêdog County has stimulated rapid change and possible loss of traditional knowledge. The influence of modernization, social and economic development, and the lack of interests shown by the young generation are seriously threatened to the ethnic culture of no written words [89]. Our research shows that the increasing publicity and availability of Tibetan and Chinese medicines has also affected the indigenous knowledge of the Monpa. There are no exclusive traditional doctors in the villages now and traditional medical knowledge is about to disappear.

## Conclusions

Monpa traditional plant-based knowledge are practiced, accumulated, and passed down from generation to generation. The Monpa people in Mêdog County still preserve most of traditional plant-based knowledge. We documented 194 wild plant species belonging to 82 families 158 genera used for traditional medicines, food, dyeing, timber, religion, and other purposes during our ethnobotanical survey. Overall, this study provides a deeper understanding of the Monpa traditional knowledge on wild plants. The study suggests some wild medical plant species might have new active ingredients which are necessitated for further investigation. Since the development of modernization has changed the Monpa lifestyle and production structure, traditional knowledge and biocultural diversity can be essential components to ensure the sustainable development of Monpa community and may play a significant role in the sustainable use and development of Tibetan plant resources.

### Supplementary information

**Additional file 1.** The Tibetan alphabet (Consonants).

**Additional file 2.** The Tibetan alphabet (Vowels).

## Data Availability

All data generated or analysed during this study are included in this published article and its supplementary information files.

## Appendix

**Table 4:** Ethnobotanical inventory of Monpa in Mêdog County, Tibet, China

Family name	Scientific name	Vernacular name	Habit	Parts used	Local use	f	CI	Voucher specimen number
Acanthaceae	*Strobilanthes cusia* (Nees) Kuntze	Yang-shar-pa	Herb	Leaves	Dye plant	0.25	0.25	WangYH0009
Actinidiaceae	*Saurauia punduana* Wall.	A-rong-ma	Tree	Flower buds and fruits	Food (a kind of fruit)	0.78	0.78	18CS16811
Adoxaceae	*Sambucus williamsii* Hance	Bha-mu-klung-shi	Shrub	Leaves	Leaves are baked on the fire with butter used for treating bruises			18CS16839
Adoxaceae	*Viburnum cylindricum* Buch.-Ham. ex D. Don	Uh-mu-ling-shing	Tree	Fruits and burgeons	Fruit oil soaked in alcohol for anti-inflammatory and soaked in honey for removing scar. Burgeons are boiled in water for repelling flea	0.05	0.05	18CS16894
Adoxaceae	*Viburnum erubescens* Wall.	Tseh-za-klu-shing	Tree	Fruits	Food (a kind of fruit)			18CS16949
Altingiaceae	*Altingia excelsa* Noronha	Sang-shing	Tree	Branches, fruits and burgeons	Fuel. Fruits are burned for refreshing. Making tea	0.53	0.53	18CS16869
Amaranthaceae	*Chenopodium album* L.	Shar-ri-mu	Herb	Seeds	Food (extracting starch)	0.09	0.09	18CS16857
Anacardiaceae	*Choerospondias axillaris* (Roxb.) B. L. Burtt & A. W. Hill	Ju-ru-ra	Tree	Fruits	Food (a kind of fruit)	0.16	0.16	18CS16940
Apiaceae	*Pimpinella diversifolia* DC.	Gya-ma-ga-dsa	Herb	Whole plant	Food (a kind of vegetable)	0.56	0.56	18CS16816
Apocynaceae	Apocynaceae sp.	Nu-ru	Vine	Roots and stems	Medicine used for treating allergy			18CS16899
Araceae	*Alocasia longiloba* Miq.	Bo-zong-gang-gyi-pa	Herb	Roots	Sliced roots used for anti-infective and treating burns			WangYH0069
Araceae	*Colocasia affinis* Schott	Bu-dong	Herb	Rhizomes	Food (a kind of vegetable). Extracting starch	0.17	0.17	18CS16922
Araceae	*Colocasia antiquorum* Schott	Bu-rong	Herb	Whole plant	Food (a kind of vegetable). Extracting starch	0.11	0.11	18CS16824
Araceae	*Colocasia esculenta* (L.) Schott	Pon-song	Herb	Whole plant	Food (a kind of vegetable). Extracting starch	0.05	0.05	WangYH0184
Araceae	*Remusatia pumila* (D. Don) H. Li & A. Hay	Pon-song	Herb	Whole plant	Food (a kind of vegetable, and boiled in the water)	0.05	0.05	WangYH0029
Araceae	*Remusatia vivipara* (Roxb.) Schott	Ri-bo-srong	Herb	Leaves	Food (a kind of vegetable, and boiled in the water)			WangYH0178
Araceae	*Sauromatum venosum* (Dryand. ex Aiton) Kunth	Reh-drong-ma	Herb	Whole plant	Crushed plants are used for treating suppuration			18CS16876
Araliaceae	*Brassaiopsis hainla* (Buch.-Ham.) Seem.	Bhong-dong-shing	Tree	Barks	Boiled for treating arthritis	0.05	0.05	18CS16883
Araliaceae	*Hydrocotyle javanica* Thunb.	Sa-la-meng-ba-ren	Herb	Whole plant	Fish poison plant	0.06	0.06	18CS16948
Araliaceae	*Schefflera khasiana* (C. B. Clarke) R. Vig.	Pyu-shing	Tree	Stems	Musical instrument	0.22	0.22	18CS16897
Arecaceae	*Arenga micrantha* C. F. Wei	Ta-shi	Tree	Stems	Food (extracting starch). Forage	0.34	0.36	18CS16836
Arecaceae	*Arenga pinnata* (Wurmb) Merr.	Ta-shing	Tree	Stems	Extracting starch	0.16	0.16	WangYH0132
Arecaceae	*Calamus acanthospathus* Griff.	B-nyu-mu/ Ba-ser	Vine	Burgeons and fruits	Food (a kind of vegetable and fruit). Making agriculture tools	0.43	0.43	18CS16864
Arecaceae	*Caryota obtusa* Griff.	Chu-shing	Tree	Stems	Making chopsticks	0.09	0.09	18CS16819
Asparagaceae	*Polygonatum oppositifolium* (Wall.) Royle	Ren-gyi-tsong	Herb	Burgeons	Food (a kind of vegetable)	0.05	0.05	WangYH0194
Asparagaceae	*Rohdea nepalensis* (Raf.) N.Tanaka	Ka-lu	Herb	Tender stems	Food (a kind of vegetable)			18CS16958
Athyriaceae	*Diplazium esculentum* (Retz.) Sw.	Ta-wai	Fern	Tender stems and leaves	Food (a kind of vegetable)	0.34	0.34	18CS16817
Balsaminaceae	*Impatiens arguta* Hook.f. & Thomson	Gyang-tsong-hwen	Herb	Leaves	Crushed leaves are used for stopping bleeding. Forage	0.33	0.33	WangYH0050
Begoniaceae	*Begonia aborensis* Dunn	Gyu-bu	Herb	Stems	Food (a kind of vegetable)	0.08	0.08	18CS16834
Begoniaceae	*Begonia acetosella* Craib	Pa-pa-man	Herb	Leaves	Mashed leaves are used for treating leeches bite			18CS16936
Berberidaceae	*Holboellia latifolia* Wall.	Chou-dang-lie-si	Vine	Fruits	Food (a kind of fruit)	0.2	0.2	18CS16898
Bignoniaceae	*Radermachera yunnanensis* C.Y.Wu	Gya-srong-ni-shing	Tree	Stems	Making agriculture tools	0.08	0.08	18CS16944
Boraginaceae	*Cordia dichotoma* G. Forst.	Pa-mi-shing	Tree	Fruits	Extracting oil	0.13	0.13	18CS16818
Brassicaceae	*Cardamine macrophylla* Willd.	Shu	Herb	Whole plant	Food (a kind of vegetable)			18CS16852
Brassicaceae	*Rorippa dubia* (Pers.) H.Hara	Ling-zong-mer-mu	Herb	Whole plant	Food (a kind of vegetable)	0.31	0.31	18CS16835
Campanulaceae	*Codonopsis affinis* Hook.f. & Thomson	Gyang-phu-sen	Herb	Fruits	Spice plant	0.27	0.27	WangYH0037
Campanulaceae	*Codonopsis inflata* Hook.f.	Gyang-hu-ser	Vine	Fruits	Spices plant	0.05	0.05	WangYH0038
Cannabaceae	*Celtis tetrandra* Roxb.	Long-shing	Tree	Fruits	Food (a kind of fruit)	0.2	0.2	18CS16903
Caprifoliaceae	*Leycesteria formosa* Wall.	Pya-min-mon	Shrub	Leaves	Medicine uesd for stopping bleeding			WangYH0170
Celastraceae	*Celastrus glaucophyllus* Rehder & E. H. Wilson	Ling-shing	Twining shrub	Trunks	Timber plant			WangYH0048
Clusiaceae	*Garcinia nujiangensis* C. Y. Wu & Y. H. Li	La-ga-dong-shing	Tree	Fruits and stems	Food (a kind of fruit). Fuel			18CS16931
Clusiaceae	*Garcinia pedunculata* Roxb. ex Buch.-Ham.	Kor-mang	Tree	Fruits	Food (a kind of fruit)			WangYH0027
Combretaceae	*Terminalia myriocarpa* Van Heurck & Müll. Arg.	Ba-lem-shing	Tree	Trunks	Timber plant			WangYH0124
Commelinaceae	*Streptolirion volubile* Edgew.	Pa-ner-ju	Herb	Whole plant	Forage			18CS16885
Compositae	*Acmella oleracea* (L.) R. K. Jansen	Nyi-ra-ki	Herb	Burgeons	Food (a kind of vegetable)	0.13	0.13	WangYH0056
Compositae	*Artemisia vestita* Wall. ex Besser	Myer-rang-ma	Herb	Leaves	The powder of “xin zei” are wrapped in leaves and rolled up, and then placed on the navel as moxibustion, used for treating stomach pain			18CS16868
Compositae	*Bidens pilosa* L.	Sgrong-treng-rong	Herb	Leaves	Boiled leaves used for treating cold, sore throat and stuffy nose	0.05	0.05	18CS16872
Compositae	*Crassocephalum crepidioides* (Benth.) S. Moore	Gyal-pa-ehn	Herb	Whole plant	Food (a kind of vegetable)	0.78	0.78	18CS16809
Compositae	*Gynura procumbens* (Lour.) Merr.	Wen-gya-pa	Herb	Aerial parts	Forage	0.06	0.06	WangYH0121
Compositae	*Helianthus tuberosus* L.	Yang-gyal	Herb	Tubers				18CS16854
Cornaceae	*Cornus capitata* Wall.	Da-ming-der-shing	Tree	Fruits	Food (a kind of fruit)	0.08	0.08	18CS16950
Cucurbitaceae	Cucurbitaceae sp.	Doe-shung	Vine	Tubers	Washing hair and clothes	0.19	0.19	18CS16823
Cucurbitaceae	*Momordica dioica* Roxb. ex Willd.	Su-ba	Herb	Leaves	Food (a kind of vegetable), boiled leaves used for treatinng cholecystitis. Washing hair and clothes	0.31	0.31	18CS16874
Cucurbitaceae	*Solena heterophylla* Lour.	Gang-gu-long	Herb	Fruits	Food (a kind of fruit)	0.25	0.25	18CS16873
Cucurbitaceae	*Thladiantha cordifolia* (Blume) Cogn.	Su-pa	Vine	Tubers	Washing hair and clothes	0.34	0.34	18CS16863
Cucurbitaceae	*Trichosanthes tricuspidata* Lour.	A-pa-kas	Vine	Seeds	Food (a kind of vegetable)	0.06	0.06	18CS16943
Cucurbitaceae	*Zehneria japonica* (Thunb.) H.Y. Liu	Ka-gyi	Herb	Whole plant	Food (a kind of vegetable)	0.19	0.19	18CS16826
Cupressaceae	*Platycladus orientalis* (L.) Franco	shug-pa	Tree	Stems	Religious ritual use			WangYH0017
Cyatheaceae	*Alsophila articulata* J. Sm. ex T. Moore & Houlston	A-gyi	Tree	Stems	Making alcohol beverages. Extracting starch	0.16	0.18	WangYH0108
Cyperaceae	*Scirpus rosthornii* Diels	Gong-bu-ueh	Herb	Fruits	Food (a kind of fruit)	0.06	0.06	18CS16946
Dioscoreaceae	*Dioscorea alata* L.	Dgro-ton/ Gyu-dang	Vine	Rhizomes	Food (a kind of vegetable). Extracting starch	0.08	0.08	WangYH0140
Dioscoreaceae	*Dioscorea melanophyma* Prain & Burkill	Bo-zon-za-lu	Vine	Leaves and rhizomes	Food (a kind of vegetable). Extracting starch. Forage	0.06	0.06	WangYH0059
Dioscoreaceae	*Dioscorea pentaphylla* L.	Pan-dang	Vine	Roots	Extracting starch. Forage. Food (a kind of fruit)	0.34	0.34	18CS16889
Dioscoreaceae	*Dioscorea* sp.	Ju-dang	Vine	Rhizomes	Food (a kind of vegetable). Extracting starch	0.36	0.36	18CS16877
Ebenaceae	*Diospyros lotus* L.	A-mu-dong-ba-shing	Tree	Fruits	Food (a kind of fruit)			18CS16954
Ebenaceae	*Diospyros variegata* Kurz	Ang-dri-pha	Shrub	Fruits	Food (a kind of fruit)			WangYH0104
Elaeagnaceae	*Elaeagnus conferta* Roxb.	Trong-pa-lin	Shrub	Fruits	Food (a kind of fruit)			18CS16952
Elaeagnaceae	*Elaeagnus umbellata* Thunb.	Dar-ma	Shrub	Fruits	Food (a kind of fruit)	0.27	0.27	18CS16859
Elaeocarpaceae	*Elaeocarpus braceanus* Watt ex C. B. Clarke	Gar-shar-dong-shing	Tree	Seeds and fruits	Boiled seeds used for treating diarrhea. Food (a kind of fruit)	0.5	0.5	18CS16858
Elaeocarpaceae	Elaeocarpaceae sp.	Dang-bu-ru	Tree or shrub	Fruits	Extracting oil	0.19	0.19	18CS16942
Equisetaceae	*Equisetum ramosissimum* Desf.	Nyer-tshyu	Herb	Roots and aerial parts	Food (a kind of fruit). Boiled liquid for treating rheumatism	0.3	0.3	18CS16878
Ericaceae	*Craibiodendron henryi* W. W. Sm.	Shar-kor-shing	Tree	Branches and leaves	Put in the field for treating rice blast			18CS16890
Ericaceae	*Gaultheria straminea* R. C. Fang	Tsong-pa-ling	Shrub	Fruits	Food (a kind of fruit)	0.08	0.08	18CS16861
Euphorbiaceae	*Macaranga denticulata* (Blume) Müll.Arg.	Tsa-la-ga	Tree	Leaves	Making agriculture tools			18CS16909
Euphorbiaceae	*Ostodes paniculata* Blume	Ga-ren-de-shing	Tree	Seeds	Extracting oil	0.06	0.06	18CS16840
Euphorbiaceae	*Ricinus communis* L.	Gyal-mu-na	Herb	Leaves	Leaves are baked on the fire with butter used for treating bruises. Seed oils			18CS16888
Fagaceae	*Castanopsis clarkei* King ex Hook.f.	Suo-na	Tree	Fruits	Nut	0.11	0.11	18CS16938
Gentianaceae	*Crawfurdia angustata* C. B. Clarke	Suo-long-ma	Herb	Leaves and flowers	Food (a kind of vegetable)	0.09	0.09	18CS16831
Gentianaceae	*Swertia angustifolia* Buch.-Ham. ex D. Don	Pau-sein-po	Herb	Leaves and roots	Medicine used for treating malaria			WangYH0023
Gentianaceae	*Swertia nervosa* (Wall. ex G. Don) C. B. Clarke	Pa-bhu-ser-pu	Herb	Leaves and roots	Leaves are boiled in the water used for treating diarrhea			18CS16841
Gnetaceae	*Gnetum pendulum* C. Y. Cheng	Gyong-ga-sa	Vine	Fruits	Nut	0.22	0.22	18CS16959
Hydrangeaceae	*Dichroa febrifuga* Lour.	Yo-gor-shing	Shrub	Branches	Branches are burned as mosquito repellent			18CS16901
Hypericaceae	*Hypericum bellum* H. L. Li	Kor-ma-shing	Shrub	Fruits	Food (sweet taste)			WangYH0110
Hypoxidaceae	*Molineria capitulata* (Lour.) Herb.	Tsan-ngan	Herb	Fruits	Food (a kind of fruit)	0.27	0.27	18CS16829
Lamiaceae	*Elsholtzia blanda* (Benth.) Benth.	Na-gang-shing	Herb	Aerial parts	Incense plant			WangYH0071
Lamiaceae	*Elsholtzia feddei* H.Lév.	Pa-pi	Herb	Leaves	Spice plant for making blood sausage			18CS16882
Lamiaceae	*Isodon lophanthoides* (Buch.-Ham. ex D.Don) H.Hara	Ra-khu-la-dang	Herb	Whole plant	Boiled liquid for treating intestinal parasites			WangYH0172
Lamiaceae	*Mosla dianthera* (Buch.-Ham. ex Roxb.) Maxim.	Shing-nang-gu-lu	Herb	Leaves	Chewed leaves used for treating allergies			18CS16871
Lamiaceae	*Perilla frutescens* (L.) Britton	Nang	Herb	Seeds	Extracting oil	0.08	0.08	WangYH0199
Lamiaceae	*Pogostemon brevicorollus* Y.Z.Sun	Na-mu-sein	Herb	Whole plant	Food (a kind of vegetable)	0.2	0.2	18CS16865
Lauraceae	*Cinnamomum contractum* H. W. Li	Shing-tsa	Tree	Roots	Crushed roots are used for stomach pain. Tobacco substitutes	0.28	0.28	18CS16892
Lauraceae	*Cinnamomum iners* Reinw. ex Blume	Lho-pa-sang-shing	Tree	Leaves	Incense plant	0.06	0.06	WangYH0030
Lauraceae	*Litsea tibetana* Yen C. Yang & P. H. Huang	Snying-shing	Shrub	Fruits	Extracting oil	0.09	0.09	18CS16934
Leguminosae	*Amphicarpaea bracteata* subsp. edgeworthii (Benth.) H.Ohashi	Shor-ru	Herb	Roots	Forage			WangYH0094
Leguminosae	*Derris scabricaulis* (Franch.) Gagnep.	Ang-du-ru	Liana	Roots	Fish poison plant	0.16	0.16	18CS16821
Leguminosae	*Erythrina arborescens* Roxb.	Tsa-shing	Tree	Stems and leaves	Stems are used for carving materials. Making agriculture tools	0.25	0.32	18CS16891
Leguminosae	*Entada rheedii* Spreng.	Kor-lo-ba-ru	Vine	Fruits	Food (remove toxicity by boiling 10 times)	0.22	0.22	18CS16846
Leguminosae	*Millettia pachycarpa* Benth.	Ngra-ru	Liana	Seeds and roots	Crushed seeds and roots are used for killing insects			WangYH0061
Loranthaceae	*Tripodanthus acutifolius* (Ruiz & Pav.) Tiegh.	Tsa-snying	Shrub parasitic	Fruits	Food (a kind of fruit)			18CS16928
Malvaceae	*Abroma augusta* (L.) L.f.	Go-men-ta-dong-shing	Shrub	Whole plant	Making agriculture tools	0.06	0.06	WangYH0003
Malvaceae	Malvaceae sp.	Pu-lang-shing	Tree	Fruits	Food (a kind of fruit)	0.06	0.06	18CS16827
Malvaceae	*Sterculia lanceifolia* Roxb.	Bha-ba-ba-gu	Tree or shrub	Fruits	Food (a kind of fruit)	0.23	0.23	18CS16910
Malvaceae	*Urena lobata* L.	Tsi-ming-uenh	Herb	Whole plant	Religious ritual use	0.22	0.22	18CS16893
Marantaceae	*Stachyphrynium placentarium* (Lour.) Clausager & Borchs.	La-gu-la-la	Herb	Leaves	Making agriculture tools			WangYH0196
Melanthiaceae	*Paris polyphylla* ssp.	A-du-ba-du	Herb	Rhizomes	Boiled liquid for treating diabetes			18CS16853
Menispermaceae	*Stephania abyssinica* (Quart.-Dill. & A.Rich.) Walp.	Ru-dour	Woody vine	Roots	Boiled the dried roots used for treating rheumatism and snake bite			WangYH0177
Menispermaceae	*Stephania* sp.	Yong-ju-pin	Woody vine	Fruits	Food (a kind of fruit)	0.22	0.22	18CS16932
Moraceae	*Ficus auriculata* Lour.	Ba-drong-ma-shing	Tree	Fruits and leaves	Food (a kind of fruit). Forage	0.06	0.08	18CS16960
Moraceae	*Ficus cyrtophylla* (Wall. ex Miq.) Miq.	Pa-ju-ma	Tree or shrub	Fruits	Beverage			18CS16915
Moraceae	*Ficus oligodon* Miq.	Ba-ler-drong-ma	Tree	Fruits	Food (a kind of fruit)	0.09	0.09	18CS16918
Moraceae	*Ficus semicordata* Buch.-Ham. ex Sm.	Drong-ma	Tree	Fruits and leaves	Fruits are eaten directly. Leaves are used as sandpaper to burnish the bowl	0.39	0.43	18CS16832
Moraceae	*Ficus subincisa* Buch.-Ham. ex Sm.	Rel-me-sgrong-ma	Tree	Fruits	Food (a kind of fruit)			18CS16925
Moraceae	*Morus alba* L.	Sems-ling-shing	Tree	Fruits	Food (a kind of fruit)	0.17	0.17	18CS16902
Moraceae	*Morus wittiorum* Hand.-Mazz.	Sems-ling-shing	Tree	Stems	Boiled liquid for treating leprosy. Timber plant	0.25	0.25	18CS16947
Musaceae	*Musa sanguinea* Hook.f.	A-nyi-lae-sih	Herb	Fruits	Food (a kind of fruit)	0.3	0.3	WangYH0176
Nephrolepidaceae	*Nephrolepis cordifolia* (L.) C. Presl	Ta-wai	Fern	Fruits	Food (a kind of fruit)	0.33	0.33	18CS16896
Oleaceae	*Fraxinus floribunda* Wall.	Tra-per-shing	Tree	Barks	Boiled liquid for treating sprain and sunburn			18CS16912
Omphalotaceae	*Lentinus sajor-caju* Fr.	Bren-ba-ba-mu	Fungi	Mushroom	Food (a kind of vegetable)			18CS16933
Omphalotaceae	*Lentinus* sp.	Tsyer-gen-ba-mu	Fungi	Mushroom	Food (a kind of vegetable)			18CS16930
Ophioglossaceae	*Ophioglossum vulgatum* L.	Gu-gu-meng	Grass	Burgeons	Food (a kind of vegetable)	0.06	0.06	18CS16844
Orchidaceae	*Dendrobium catenatum* Lindl.	Shi-hu	Herb epiphytic	Stems	Boiled liquid for treating cold			18CS16856
Oxalidaceae	*Oxalis corniculata* L.	Ju-bu-uenh	Herb	Leaves	Eaten directly, used for treating morning sickness			18CS16867
Pentaphylacaceae	*Eurya acuminata* DC.	Zem-shing	Tree or shrub	Leaves	Dye plant and mordant			WangYH0067
Phytolaccaceae	*Phytolacca acinosa* Roxb.	Mye-mye-gang-pu-mon	Herb	Leaves	Spice plant	0.06	0.06	18CS16881
Pinaceae	*Pinus wallichiana* A.B.Jacks.	Shog-shing-nang	Tree	Trunks	Timber plant	0.2	0.2	WangYH0128
Piperaceae	*Piper semiimmersum* C. DC.	Pi-pi-ling	Climber	Leaves	Boiled the dried leaves used for treating altitude sickness and irregular menstruation			18CS16845
Piperaceae	*Piper* sp.	Sa-pa	Shrub or climber	Leaves	Mashed leaves used for stopping bleeding			18CS16822
Piperaceae	*Piper sylvaticum* Roxb.	Pang-ser	Climber	Leaves	Mashed leaves used for anti-inflammatory			WangYH0188
Poaceae	*Arundo donax* L.	Sra-gu	Bamboo	Leaves and stems	Fuel			WangYH0097
Poaceae	*Bambusa teres* Munro	Li-shing	Bamboo	Culms	Making bow and arrow	0.09	0.09	WangYH0092
Poaceae	*Coix lacryma-jobi* L.	Phon-pa-lin	Herb	Seeds	Boiled liquid for treating high blood pressure. Ornament plant	0.27	0.27	WangYH0179
Poaceae	*Dendrocalamus tibeticus* Hsueh & T. P. Yi	Ha-po	Bamboo	Culms and shoots.	Food (a kind of vegetable). Making agriculture tools	0.1	0.1	WangYH0160
Poaceae	*Eleusine coracana* (L.) Gaertn.	Kon-pu	Herb	Seeds	Making alcohol beverages. Extracting starch	0.47	0.5	WangYH0001
Poaceae	*Imperata cylindrica* (L.) Raeusch.	Shing-pu	Herb	Leaves	Thatching	0.06	0.06	18CS16843
Poaceae	*Phyllostachys mannii* Gamble	Suo-nong	Bamboo	Burgeons	Food (a kind of vegetable)	0.08	0.08	18CS16907
Poaceae	*Sorghum bicolor* (L.) Moench	Phin-nang	Herb	Stems and seeds	Stems are eaten directly. Seeds are used for preparing alcohol beverages	0.05	0.12	18CS16814
Poaceae	*Themeda villosa* (Lam.) A.Camus	Pi-li	Herb	Leaves	Thatching	0.11	0.11	18CS16842
Polygonaceae	*Fagopyrum acutatum* (Lehm.) Mansf. ex K.Hammer	Pin-dae-mu	Herb	Leaves	Food (a kind of vegetable). Forage	0.13	0.15	18CS16813
Polygonaceae	*Fagopyrum esculentum* Moench	Ka-la	Herb	Fruits	Food (extracting starch)	0.13	0.13	18CS16855
Polygonaceae	*Fagopyrum tataricum* (L.) Gaertn.	Ka-la	Herb	Seeds	Making alcohol beverages. Extracting starch	0.17	0.21	WangYH0182
Polygonaceae	*Persicaria capitata* (Buch.-Ham. ex D.Don) H.Gross	Long-pa-dang-mon-nang	Herb	Whole plant	Medicine used for burns	0.05	0.05	WangYH0155
Polygonaceae	*Persicaria nepalensis* (Meisn.) Miyabe	Gong-sgrer-ming	Herb	Whole plant	Food (a kind of fruit). Forage	0.06	0.08	18CS16919
Polygonaceae	*Polygonum chinense* var. ovalifolium Meisner	Gu-ju-ma-shing	Herb	Tender stems and leaves	Food (a kind of vegetable)	0.09	0.09	18CS16820
Polyporaceae	*Polyporus* sp.	Shing-pa-mu	Fungi	Mushroom	Food (a kind of vegetable)	0.09	0.09	18CS16917
Primulaceae	*Embelia floribunda* Wall.	Ju-bu-ru	Vine	Fruits and roots	Food (a kind of fruit)	0.09	0.09	18CS16927
Primulaceae	*Maesa marioniae* Merr.	Ker-seh-ru	Shrub	Fruits	Food (a kind of fruit)			18CS16953
Primulaceae	*Maesa rugosa* C. B. Clarke	Lho-ku-mer-shing	Shrub	Leaves	Making agriculture tools	0.09	0.09	18CS16957
Ranunculaceae	*Clematis napaulensis* DC.		Vine	Leaves	Forage			WangYH0058
Rhamnaceae	*Hovenia acerba* Lindl.	Shi-pi	Tree	Fruits	Boiled or eaten directly, used for alcoholism			18CS16884
Rhamnaceae	*Rhamnus napalensis* (Wall.) M. A. Lawson	Da-gor-shing	Shrub	Fruits	Food (a kind of fruit)	0.06	0.06	18CS16923
Rosaceae	*Chaenomeles cathayensis* (Hemsl.) C. K. Schneid.	Tong-ju-bha-bu	Tree	Fruits	Food (a kind of fruit)	0.27	0.27	18CS16921
Rosaceae	*Duchesnea indica* (Jacks.) Focke	Pu-tshu-la-gong	Herb	Fruits	Food (a kind of fruit), boiled the dried fruits used for detoxification and treating bruises	0.41	0.41	18CS16875
Rosaceae	*Laurocerasus undulata* (Buch.-Ham. ex D. Don) M. Roemer	Dan-bur	Tree or shrub	Seeds	Extracting oil. Religious ritual use			WangYH0068
Rosaceae	*Prunus persica* (L.) Batsch	Lin-shing	Tree	Fruits	Food (a kind of fruit)	0.33	0.33	18CS16956
Rosaceae	*Rubus ellipticus* Sm.	Tser-gong	Shrub	Fruits	Food (a kind of fruit)	0.33	0.33	18CS16850
Rosaceae	*Rubus niveus* Thunb.	Tu-lu-tse-gong	Shrub	Fruits	Food (a kind of fruit)	0.05	0.05	18CS16815
Rosaceae	*Rubus sumatranus* Miq.	Ga-bu-dong-tse-gong	Shrub	Fruits	Food (a kind of fruit)	0.14	0.14	18CS16939
Rubiaceae	*Luculia gratissima* (Wall.) Sweet	Nom-meng	Tree or shrub	Flowers	Religious ritual use			WangYH0051
Rubiaceae	*Mussaenda pubescens* Dryand.	Meng-gya-bai-dong-shing	Shrub	Leaves	Seasonal indication	0.06	0.06	18CS16941
Rubiaceae	*Ophiorrhiza medogensis* H. Li	Ming-zi-ma-mu	Herb	Whole plant	Food (a kind of vegetable)	0.11	0.11	18CS16862
Rubiaceae	*Rubia membranacea* Diels	Lae-nyi	Herb	Stems	Dye plant			WangYH0114
Rubiaceae	*Rubia wallichiana* Decne.	Lae-nyi	Herb	Stems	Dye plant	0.3	0.3	WangYH0127
Rubiaceae	*Spiradiclis* sp.	Mi-zu-ma	Herb	Whole plant	Food (a kind of vegetable)	0.09	0.09	18CS16833
Rubiaceae	*Uncaria rhynchophylla* (Miq.) Miq. ex Havil.	Gou-du	Liana	Stems	Boiled liquid for treating high blood pressure			18CS16962
Rubiaceae	*Uncaria scandens* (Sm.) Hutch.	Tsae-tsu	Liana	Leaves	Boiled liquid for treating epilepsy			18CS16905
Rubiaceae	*Wendlandia tinctoria* (Roxb.) DC.	Mehi-neng-nang-shi	Tree or shrub	Stems	Making agriculture tools	0.17	0.17	18CS16900
Rutaceae	*Citrus medica* L.	Hpo-rang-nying-pa	Tree or shrub	Fruits	Medicine used for treating cold			
Rutaceae	*Toddalia asiatica* (L.) Lam.	Ae-pi-ka-ba	Shrub	Seeds	Seed oil with butter is used for treating epilepsy			18CS16961
Rutaceae	*Zanthoxylum motuoense* C. C. Huang	Gei	Tree	Fruits	Crushed fruits are used for treating bad skin odour. Spice plant	0.56	0.56	18CS16895
Sabiaceae	*Meliosma pinnata* (Roxb.) Maxim.	Beng-shar-shing	Tree	Flowers	Seasonal indication	0.06	0.06	18CS16945
Scrophulariaceae	*Buddleja asiatica* Lour.	Yang-ren	Shrub	Whole plant	Making alcohol beverages			18CS16914
Solanaceae	*Datura stramonium* L.	Yun-ma-chu-dong	Herb or subshrub	Seeds	Medicine used for treating toothache	0.06	0.06	18CS16837
Solanaceae	*Nicotiana tabacum* L.	Da-mu-ga	Herb	Leaves	Crushed leaves used for treating sinusitis			18CS16906
Solanaceae	*Solanum americanum* Mill.	Gu-ju-shu	Herb	Burgeons	Food (a kind of vegetable)	0.14	0.14	18CS16838
Solanaceae	*Solanum aculeatissimum* Jacq.	Kha-lang-gyi	Herb to subshrub	Roots	Crushed roots and the leaves of *Luffa cylindrica* are used for treating psoriasis			18CS16866
Solanaceae	*Solanum torvum* Sw.	Kha-lang-gyi	Shrub	Fruits	Food (a kind of vegetable)	0.2	0.2	18CS16812
Taxaceae	*Torreya grandis* var. *yunnanensis* (W.C.Cheng & L.K.Fu) Silba	Gae-long-shing	Tree	Fruits	Food (a kind of fruit)			18CS16880
Thymelaeaceae	*Edgeworthia gardneri* (Wall.) Meisn.	Sho-gu-shing/ Ju-pu -shing	Tree	Barks	Papermaking	0.05	0.05	WangYH0006
Urticaceae	*Debregeasia longifolia* (Burm.f.) Wedd.	Rang-shing	Shrub	Roots	Boiled liquid for preventing miscarriage and treating bruises			18CS16870
Urticaceae	*Elatostema cuneiforme* W.T.Wang	Tsen-tsen-pa	Herb	Aerial parts	Forage	0.08	0.08	WangYH0091
Urticaceae	*Elatostema nasutum* Hook.f.	Da-mi-ru	Herb	Leaves	Food (a kind of vegetable boiled in the water first)			18CS16848
Urticaceae	*Gonostegia hirta* (Blume ex Hassk.) Miq.	Ro-gyi-ba	Herb	Whole plant	Food (a kind of vegetable)	0.08	0.08	18CS16924
Urticaceae	*Pilea hilliana* Hand.-Mazz.	Ru-gong-su-gang	Herb	Leaves	Food (a kind of vegetable)	0.05	0.05	WangYH0159
Urticaceae	*Poikilospermum lanceolatum* (Trécul) Merr.	Ba-mi-ru	Shrub	Leaves and aerial roots	Leaves are used for forage. Aerial roots are used for rope	0.34	0.36	18CS16830
Urticaceae	*Urtica mairei* H. Lév.	Gang-dang-gyal-zu	Herb	Leaves	Food (a kind of vegetable)			WangYH0040
Violaceae	*Viola* sp.	Pian-mier	Herb	Whole plant	Boiled liquid for clearing heat and detoxification			WangYH0066
Vitaceae	*Tetrastigma serrulatum* (Roxb.) Planch.	Ju-bae-ru	Liana	Fruits	Food (a kind of fruit)	0.28	0.28	18CS16828
Xanthorrhoeaceae	*Hemerocallis fulva* (L.) L.	Chu-ta	Herb	Leaves and flowers	Food (a kind of vegetable)	0.17	0.17	18CS16887
Zingiberaceae	*Alpinia bambusifolia* C. F. Liang & D. Fang	Tar-gang	Herb	Flower buds	Food (sweet taste)	0.2	0.2	WangYH0101
Zingiberaceae	*Alpinia malaccensis* (Burm.f.) Roscoe	Tar-gang	Herb	Flower buds	Food (sweet taste)	0.2	0.2	WangYH0103
Zingiberaceae	*Curcuma aromatica* Salisb.	Dgrong	Herb	Roots and leaves	Boiled roots used for treating heat stroke, irregular menstruation and boiled leaves used for treating alcoholism			18CS16879
Zingiberaceae	*Curcuma longa* L.	Dgrong	Herb	Roots	Dye plant	0.22	0.22	WangYH0070
Zingiberaceae	*Hedychium coccineum* Buch.-Ham. ex Sm.	Ma-mi-niu-mu	Herb	Burgeons	Food (a kind of vegetable)	0.05	0.05	18CS16849
Zingiberaceae	*Hornstedtia tibetica* T.L.Wu & S.J.Chen	Su-mi	Herb	Fruits	Food (a kind of fruit)	0.08	0.08	18CS16847

Voucher specimen number with CS means collection section
