# Supplementary figures and images for: Monpa, memory, and change: an ethnobotanical study of plant use in Mêdog County, South-east Tibet, China
Source: J Ethnobiol Ethnomed. 2020 Jan 30;16:5. doi: 10.1186/s13002-020-0355-7 (PMC6993401; doi:10.1186/s13002-020-0355-7)

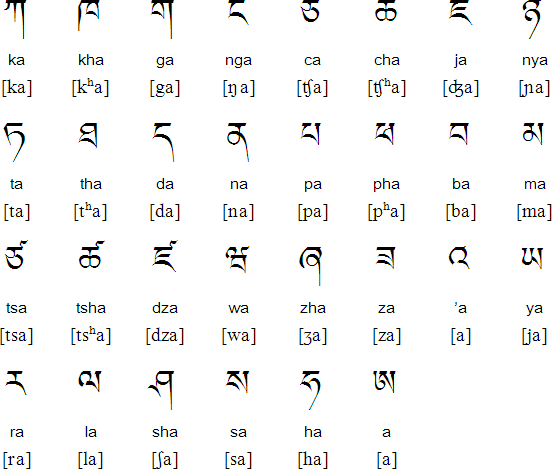

Supplement: Supplementary file 1 — Additional file 1. The Tibetan alphabet (Consonants). [file 13002_2020_355_MOESM1_ESM.gif]

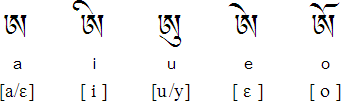

Supplement: Supplementary file 2 — Additional file 2. The Tibetan alphabet (Vowels). [file 13002_2020_355_MOESM2_ESM.gif]
